# Generating Chromosome Geometries in a Minimal Cell From Cryo-Electron Tomograms and Chromosome Conformation Capture Maps

**DOI:** 10.3389/fmolb.2021.644133

**Published:** 2021-07-22

**Authors:** Benjamin R. Gilbert, Zane R. Thornburg, Vinson Lam, Fatema-Zahra M. Rashid, John I. Glass, Elizabeth Villa, Remus T. Dame, Zaida Luthey-Schulten

**Affiliations:** ^1^ Department of Chemistry, University of Illinois at Urbana-Champaign, Urbana, IL, United States; ^2^ Division of Biological Sciences, University of California San Diego, San Diego, CA, United States; ^3^ Leiden Institute of Chemistry, Leiden University, Leiden, Netherlands; ^4^ Center for Microbial Cell Biology, Leiden University, Leiden, Netherlands; ^5^ Synthetic Biology Group, J. Craig Venter Institute, La Jolla, CA, United States

**Keywords:** cryo-electron tomography, chromosome conformation capture (3C) maps, computational modeling, whole-cell models, chromosome modeling, ribosome distribution, bacterial minimal cell, JCVI-syn3A

## Abstract

JCVI-syn3A is a genetically minimal bacterial cell, consisting of 493 genes and only a single 543 kbp circular chromosome. Syn3A’s genome and physical size are approximately one-tenth those of the model bacterial organism *Escherichia coli*’s, and the corresponding reduction in complexity and scale provides a unique opportunity for whole-cell modeling. Previous work established genome-scale gene essentiality and proteomics data along with its essential metabolic network and a kinetic model of genetic information processing. In addition to that information, whole-cell, spatially-resolved kinetic models require cellular architecture, including spatial distributions of ribosomes and the circular chromosome’s configuration. We reconstruct cellular architectures of Syn3A cells at the single-cell level directly from cryo-electron tomograms, including the ribosome distributions. We present a method of generating self-avoiding circular chromosome configurations in a lattice model with a resolution of 11.8 bp per monomer on a 4 nm cubic lattice. Realizations of the chromosome configurations are constrained by the ribosomes and geometry reconstructed from the tomograms and include DNA loops suggested by experimental chromosome conformation capture (3C) maps. Using ensembles of simulated chromosome configurations we predict chromosome contact maps for Syn3A cells at resolutions of 250 bp and greater and compare them to the experimental maps. Additionally, the spatial distributions of ribosomes and the DNA-crowding resulting from the individual chromosome configurations can be used to identify macromolecular structures formed from ribosomes and DNA, such as polysomes and expressomes.

## 1 Introduction

JCVI-syn3A is a genetically minimal bacterial cell, consisting of 493 genes and a single short 543 kbp circular chromosome derived from a Gram-positive bacterium, *Mycoplasma mycoides*. Previous work established genome-scale gene essentiality and proteomics data along with its essential metabolic network (Hutchison et al., 2016; Breuer et al., 2019) and a kinetic model of genetic information processing (Thornburg et al., 2019). The kinetic model of genetic information processes of DNA replication, transcription, and translation requires positions and length of genes along the chromosome. Spatially resolving and solving these kinetic models require cellular architecture, including spatial distributions of ribosomes and the circular chromosome. The first iteration of a bacterial cell with a synthetic genome was JCVI-syn1.0, whose synthesized genome is 1,079 kbp (Gibson et al., 2010). Syn1.0 has a doubling time of approximately 60 min and shows a spherical morphology with a radius of ∼200 nm (Hutchison et al., 2016). Two additional cycles of targeted genomic reduction resulted in JCVI-syn3.0, a cell whose synthetic genome is only 531 kbp but still autonomously replicates (Hutchison et al., 2016). Syn3.0 has a slower growth rate than Syn1.0, with a doubling time of approximately 180 min (Hutchison et al., 2016), and based on optical and scanning electron microscopy (SEM), Syn3.0 exhibits a pleomorphic morphology with significant variations (Hutchison et al., 2016; Pelletier et al., 2021). The organism that is the subject of this study, Syn3A, was created from Syn3.0 through the addition of 19 genes present in Syn1.0. While this addition made a less minimal genome, it resulted in cells with a robust spherical morphology and an average doubling time of approximately 110 min (Breuer et al., 2019). Super-resolution fluorescence microscopy (STORM) imaging (private communication, Taekjip Ha) reveals that it recovers the spherical morphology of Syn1.0, with a radius of 200–250 nm.

To create chromosome geometries for our spatial models and subsequent simulations of gene expression and translation, we develop a method of generating self-avoiding circular chromosome configurations with a resolution of 11.8 bp per monomer on a 4 nm cubic lattice. To place the chromosome inside the cell volume, we use cryo-electron tomography (cryo-ET) to define the cell boundaries and ribosome distribution, which define the regions available to the chromosome. Cryo-ET data shows that the ribosomes appear to be nearly randomly distributed throughout the cell. In cryo-ET of bacteria, the position of the chromosome is typically determined by the absence of ribosomes. For example, the cryo-ET studies of slow-growing *Escherichia coli* show ribosomes primarily localized at the poles and along the sides of the nucleoid so that the DNA can be inferred to be confined within the enclosed nucleoid region (Roberts et al., 2011). Based on the tomograms for Syn3A presented in this study, we assume the chromosome is randomly distributed among the ribosomes. We also present experimental chromosome conformation capture (3C) maps, a technique that shows the frequency at which different regions of the chromosome are in contact with each other (Dekker et al., 2002). This map shows only a single main diagonal with some small (<4 kbp) features along it and has no other significant features.

**FIGURE 1:**
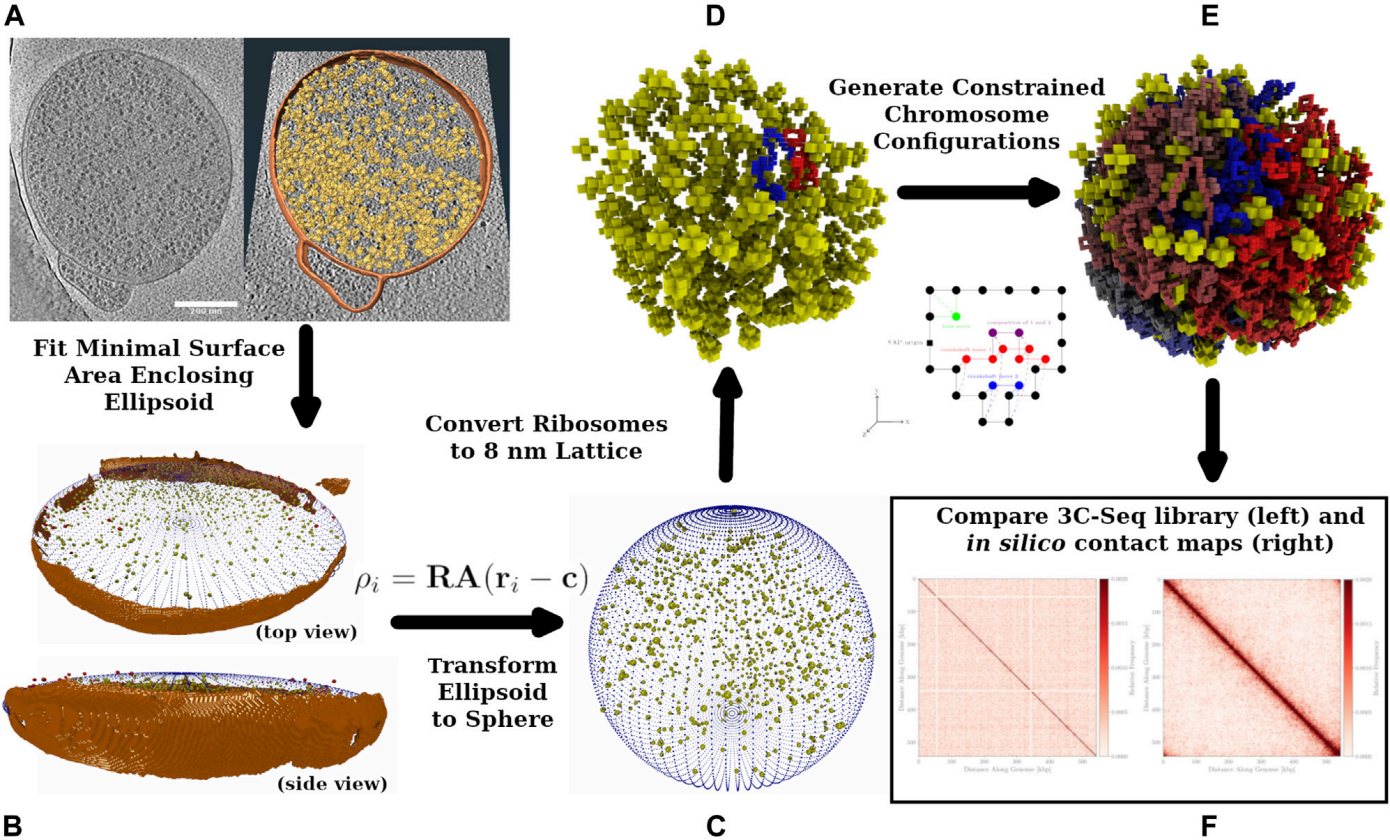
Workflow Diagram: Cryo-ET data is used to reconstruct spherical Syn3A cells, constrained chromosome configurations are generated within the reconstructed cells and resulting *in silico* chromosome contact maps are compared to the experimental 3C-Seq map. **(A)**: The left side is a single z-slice of the tomographic reconstruction. The right side are the ribosomes (yellow) identified using template-matching and the membrane segmentation (orange) superimposed on the z-slice. **(B)**: The shape of the blotted cells is approximated by an ellipsoid that is manually compared to the tomographic membrane segmentation (orange). In an iterative procedure, a series of minimal surface area enclosing ellipsoids (MSAEE) are fit around the ribosome coordinates while extraneous ribosomes assessed to be outside of the true membrane are removed within each iteration. The final fitted ellipsoid is shown in blue and the extraneous ribosomes in red. **(C)**: As Syn3A cells are known to have a spherical morphology, the ribosome coordinates and ellipsoidal membrane approximation are then transformed to a sphere with equivalent surface area. **(D)**: The continuum representation is then converted to an 8 nm cubic lattice representation used for whole-cell simulations with LM. **(E)**: Circular and self-avoiding chromosome configurations are generated as a lattice polymer on a 4 nm cubic lattice. The 4 nm lattice is coincident with the 8 nm cubic lattice and the chromosomes are constrained to avoid the ribosomes and remain within the membrane. In the representative DNA configuration, monomers are colored red and blue on opposite arms of the chromosome. **(F)**: *In silico* contact maps from ensembles of generated DNA configurations are compared to the experimental 3C-Seq map.

Using our knowledge of Syn3A’s proteome data and genome, along with the experimental cryo-ET and 3C-Seq maps, we have created a physics-based model of the chromosome to generate chromosome configurations and predict contact maps. A diagram of the workflow is presented in Figure 1 that shows the process of annotating ribosome locations and membrane from the tomograms and using the ribosome locations as constraints on chromosome configurations. The configurations are also influenced by the features present in the experimental contact map. Hi-C analysis, a variant of the chromosome conformation capture (3C) method, has been used extensively to describe the structure of eukaryotic chromatin (Lieberman-Aiden et al., 2009; van Berkum et al., 2010; Belton et al., 2012). Those chromosome contact maps are used to generate chromatin structures based on topologically associated domains (TADs) observed in the contact maps (Dekker et al., 2013; Rao et al., 2014; Fudenberg et al., 2016; Di Pierro et al., 2017). While considerations of the energy functions used in the chromatin models for eukaryotic studies are helpful in designing a bacterial study, there are bacteria-specific proteins and related effects that need to be considered when constructing a bacterial chromosome model and how the effects would appear in the resulting contact map (Le et al., 2013; Marbouty et al., 2015; Verma et al., 2019). The 3D structure of the circular bacterial chromosome at both the global and local levels is determined by effects of various nucleoid associated proteins (NAPs) and is also influenced by the crowding of ribosomes. With its reduced genome, Syn3A lacks many of the NAPs that cause significant features in the chromosome structure which leads to considerable variation from the structures and Hi-C/3C maps observed in other bacteria such as *Mycoplasma pneumoniae*, *Bacillus subtilis*, *Caulobacter crescentus*, and *Pseudomonas aeruginosa*.

The global structure of the chromosome, also known as the cellular disposition of the chromosome (Lioy et al., 2018, 2020), describes how regions of the chromosome are organized within the confines of the cell. Factors affecting the global structure include possible attachment of the chromosome to the membrane, as in *M. pneumoniae*, and loading of SMC proteins near the origin by a complete parAB*S* system, which causes alignment of the two arms of the circular chromosome (Wang et al., 2014; Wang and Rudner, 2014; Lioy et al., 2020). The parAB*S* system includes two proteins, parA and parB, which site-specifically load SMC onto *parS* sites on the DNA (Livny et al., 2007). Both of these effects result in a secondary diagonal, orthogonal to the main diagonal, in chromosome contact maps as observed in maps for *M. pneumoniae*, *B. subtilis*, *C. crescentus*, and *P. aeruginosa* (Le et al., 2013; Marbouty et al., 2015; Tran et al., 2017; Trussart et al., 2017; Lioy et al., 2020). However, the experimental contact map presented in the results section reveals that Syn3A does not have a secondary diagonal. Syn3A does not have a complete parAB*S* system because it lacks the parB protein (Breuer et al., 2019) and the complete signature *parS* sites, i.e. no sequences greater than a 10/16 match to the consensus sequence (Livny et al., 2007) were identified in a BLAST search of the genome. For comparison, when performing the same search on *B. subtilis*, we found matches of 14/16 and higher. Livny et al. also identified *Meso*, *Urea* and *Mycoplasmas* as members of the Firmicutes that lack complete parAB*S* systems (Livny et al., 2007). Therefore, we do not expect to see alignment of the two arms of the chromosome *via* the parAB*S* system and would not expect to see a secondary diagonal due to this effect. Additionally, Syn3A does not have any annotated proteins that attach the DNA to the membrane (Breuer et al., 2019), so we would not expect to see a secondary diagonal due to attachment of the DNA, unlike *M. pneumoniae* which has an attachment organelle (Trussart et al., 2017).

Factors affecting local structure include supercoiling, plectonemic loops resulting from supercoiling, small loops formed by SMC bridging distant chromosome segments, and bending and stiffening by proteins such as histone-like protein (HU), heat-stable nucleoid structuring protein (H-NS), and integration host factor (IHF) (Dame, 2005; Ohniwa et al., 2011; Dame and Tark-Dame, 2016; Dame et al., 2019; Verma et al., 2019; Birnie and Dekker, 2021). These micro level effects can strongly affect gene expression as localized crowding affects the access of the RNA polymerase (RNAP) to genes and supercoiling and plectonemes affect the RNAP’s ability to transcribe a gene (Kim et al., 2019). Of the proteins HU, IHF, and H-NS, Syn3A only has one gene, JCVISYN3A_0350, which is annotated as a putative histone-like protein with a proteomics count of 28. The count of 28 for this protein is significantly lower than the counts seen in other bacteria. For example, fast growing *E. coli* contains more than 12,000 HU (Wang et al., 2015), *B. subtilis* contains almost 9,000 HU (Wang et al., 2015), and *Mesoplasma florum* contains 9,500 HU (Matteau et al., 2020). Due to its small count, we do not expect any significant contributions to the stabilizing of chromosome loops by the protein encoded by gene JCVISYN3A_0350 and do not include it in our model.

Supercoiling is formed during transcription by the RNAP, which induces positive supercoiling in the forward direction and negative supercoiling in the reverse (Chong et al., 2014; Verma et al., 2019). Supercoiling can be eliminated by topoisomerases, gyrases, and positive/negative supercoiling annihilating each other along free DNA (Chong et al., 2014; Dorman, 2019; Verma et al., 2019). The experimental contact map presented in the Results is too sparse to distinguish between short (<10 kbp) supercoiled domains and loops and we do not see any larger interaction domains. We do not include supercoiling because of this, and given the low proteomics count of HU, we infer the DNA is in a relaxed state. As discussed above, Syn3A does not have genes that code for proteins that would attach the DNA to the cell membrane. Since the chromosome is therefore most likely not fixed at any location, we assume that, in general, DNA is mostly free, allowing positive and negative supercoiling to annihilate each other more easily. Additionally, the genome-wide proteomics counts show that we have a total of 187 RNAP and roughly 250 DNA gyrases that can alleviate positive supercoiling, 150 type IV DNA topoisomerases that can alleviate negative supercoiling, and 175 type I topoisomerases that can alleviate either (Chong et al., 2014; Breuer et al., 2019). Other bacteria are observed to have fewer topoisomerases and gyrases than RNAP, for example, fast-growing *E. coli* has roughly 3,800 topoisomerase I, 1,200 topoisomerase IV, and 6,000 to 8,000 gyrases while having over 10,000 RNAP (Bremer and Dennis, 2008; Wang et al., 2015). Another Gram-positive bacterium, *B. subtilis*, has 3,000 RNAP while only having 1,200 gyrases, 900 topoisomerase I, and 200 topoisomerase IV (Wang et al., 2015). The more closely related *M. pneumoniae* has 5,000 topoisomerase I, 200 topoisomerase IV, and 1,800 gyrases while having around 6,000 RNAP (Kühner et al., 2009). It is then more likely in these systems where larger domains have been observed in their chromosome contact maps that the proteins removing supercoiling cannot keep up with the supercoiling induced by RNAP due to their lower relative counts. Therefore, it is our assumption that as supercoiling is formed by RNAP in Syn3A, there are sufficient gyrases, topoisomerases, and negative/positive supercoiling pair annihilations to keep the DNA in a more relaxed configuration with no significant supercoiled domains.

While Syn3A does not have a complete parAB*S* system, it does contain 202 structural maintenance of chromosomes (SMC) proteins, which can bridge distant loci *via* loop extrusion powered by ATP-hydrolysis (Ganji et al., 2018; van Ruiten and Rowland, 2018). The SMC protein is a long coiled-coil protein that dimerizes and has head and hinge domains separated by approximately 50 nm (Diebold-Durand et al., 2017). The number of SMC in Syn3A is smaller than the 448 observed in *B. subtilis* (Wang et al., 2015) and 900 observed in *M. pneumoniae* (Kühner et al., 2009), but Syn3A also has a smaller volume and shorter chromosome, which could result in a higher density of loops. *M. florum* is not much larger than Syn3A and only has 85 SMCs (Matteau et al., 2020). With a higher density of SMC in both volume and chromosome length, we assume the effects of SMC looping can be significant in the chromosome structure of Syn3A. We manually annotate any of the observed regions of contact along the main diagonal in the experimental 3C-Seq map as possible loops (<4 kbp) and implement them as looping restraints in our chromosome model.

Finally, based on the cryo-ET images of Syn3A cells presented in the results, the chromosome in Syn3A is more constrained by ribosomes than in other bacteria. From the cryo-ET of Syn3A we infer that the ribosomes are uniformly distributed throughout the cells and that there is no clearly-defined condensed nucleoid region. The lack of a condensed nucleoid region is in contrast to the rod-shaped *E. coli* where the ribosomes are primarily located at the poles and along the sides of the nucleoid region (Nevo-Dinur et al., 2011; Roberts et al., 2011; Bakshi et al., 2012). We saw this distribution in cryo-ET data of slow-growing *E. coli* that was part of a previous Lattice Microbes (LM) simulation of the lac genetic switch (Roberts et al., 2011). We observe a ribosome number density of 12,920–19,370 ribosomes/μm^3^ in Syn3A cells, which is higher than the density of 4,200 ribosomes/μm^3^ in *M. pneumoniae* (Trussart et al., 2017; O’Reilly et al., 2020). The density of ribosomes in *E. coli* was previously found to be 27,000 ribosomes/μm^3^ (Bakshi et al., 2012), which is greater than the density in Syn3A, but the inferred ribosome density within the nucleoid region is 2,000–8,000 ribosomes/μm^3^. Given this, relative to other bacterial cells, the crowding of the ribosomes in Syn3A more strongly constrains the possible chromosome configurations.

In this paper, we first explain how the cellular architecture and ribosome distributions are obtained from three-dimensional cryo-electron tomograms. Using ensembles of constrained DNA configurations from our circular chromosome model on a lattice, we predict contact maps for individual cells at resolutions of 250 bp and greater and compare them to our experimental 3C-Seq map at 1,000 bp resolution. The DNA configurations in this study are generated with the intent of incorporating them into stochastic whole-cell models of Syn3A simulated using the reaction-diffusion master equation (RDME) as implemented in LM (Roberts et al., 2013; Hallock et al., 2014; Earnest et al., 2018). In the whole-cell simulations, the cellular space is divided into cubic subvolumes, so we chose to model the DNA as a lattice polymer. The DNA configurations, cell sizes, and ribosome locations presented here will later be directly incorporated into cell geometry in the kinetic simulations and will influence both diffusion and the locations at which genetic information reactions take place. We also identify potential complexes formed from ribosomes and DNA in our spatial model, such as polysomes and expressomes (O’Reilly et al., 2020), that would affect the reactions within a kinetic model.

## 2 Methods

### 2.1 Reconstructing Cell Geometries From Cryo-Electron Tomograms

#### 2.1.1 Tomogram Collection and Processing

One of the primary challenges for cellular cryo-ET is to prepare a specimen such that it is thin enough to be transparent to electrons and to vitrify thoroughly. Due to the small size of Syn3A, this can be accomplished by placing cells on a Quantifoil EM grid and blotting the majority of the liquid away followed by plunge freezing, leaving a thin layer of ice with cells embedded within it.

Initially, samples were frozen on Quantifoil Cu 200 mesh R 1/4 grids (Electron Microscopy Sciences), which have patterned holes 1 μm in diameter, with 4 μm spacing between holes (i.e., 5 μm periodicity). Our rationale was that using smaller holes would retain more Syn3A cells on the grid after blotting. However, we found that because the synthetic cells lack a cell wall, they are supple and get distorted in the direction of the flow of the medium as it is blotted away from the grid. This issue was resolved by switching to Quantifoil Cu 200 mesh R 2/1 grids, which have larger holes. Syn3A cells were grown to mid-log-phase at 37^°^C in SP4 medium (Williamson and Whitcomb, 1975) using KnockOut™ serum replacement (Invitrogen), to a density of $∼10^{8}$ cells/ml. 4 μl of sample was deposited on glow-discharged grid, blotted on the backside of the grid for 6 s using Whatman No. 1 filter paper, and plunged into a 50/50 mixture of ethane and propane (Airgas) cooled to liquid nitrogen temperatures using a manual plunger (Max Planck Institute of Biochemistry).

These *Mycoplasma* cells with synthetic genomes were more radiation sensitive than we have encountered for other bacteria. Imaging conditions were chosen to keep cumulative electron dose under 120 e/Å^2^. All data were acquired using a Titan Krios (ThermoFisher Scientific, TFS) at 300 kV and a Gatan K2 camera with a GIF energy filter, using SerialEM v3.7.4 automated protocols (Mastronarde, 2005; Schorb et al., 2019). The microscopic parameters were: 1) Pixel size: 0.53 nm (FOV: 2 μm) or 0.43 nm (FOV: 1.6 μm), 2) Target defocus: 6 μm, 3) Total accumulated dose: 90–120 e/Å^2^, 4) Tilt scheme: dose symmetric from 0^°^ to ±60^°^ every 2^°^, 5) 70 μm objective aperture. Individual tilt-series frames were aligned using MotionCor2 (Zheng et al., 2017). Tomograms were reconstructed using IMOD v4.10.29 (Kremer et al., 1996; Mastronarde, 1997; Mastronarde and Held, 2017) and binned by four for downstream template matching. Additionally, non-linear anisotropic diffusion (NAD) filtering was applied in IMOD to enhance contrast for visualization.

At the pixel size and target defocus used for acquisition, the ribosome distributions are easily discerned and can be seen for the small and large cells in Supplementary Figures S1,S2, respectively. The small cell’s dimensions and ribosome count were in good agreement with those reported previously (Hutchison et al., 2016; Breuer et al., 2019). However, the cells were flattened into ellipsoids, and sometimes further elongated. This well-known effect from blotting seems amplified in these cells due to the absence of a cell wall. The frozen cells were flattened to ∼160 nm.

To determine the ribosome distribution inside cells, we used two different approaches based on template matching, with one of them continuing to 3-D classification. First, one has to identify all ribosomes within the tomogram. Template matching is performed by creating a 3-D template of the target structure, and comparing it to each voxel in the tomogram using a 6-D search (three spatial and three rotational degrees of freedom) to identify regions that correlate highly with the template. It is noteworthy that the contrast difference between ribosomes and their surroundings in Syn3A was greatly reduced compared to other bacteria, e.g. *E. coli*, suggesting that the mass density (molecular crowding) of Syn3A is higher.

In our first approach, we used Dynamo v1.1.509 (Castaño-Díez et al., 2012) with a bacterial ribosome structure (PDB:5MDZ) as the initial template filtered to 20 Å resolution in UCSF Chimera (Pettersen et al., 2004), resampled to match the pixel size, and contrast scaled to match that of the target tomograms. A threshold cross correlation was selected so that it contained most ribosomes that were clearly inside the cell boundary. Final particle positions were inspected visually, and removed if they were membrane segments. Membranes were segmented using TomoSegMemTV (Martinez-Sanchez et al., 2014), and ribosomes outside of this segmented membrane were excluded. Starting with a high-correlation threshold, the first approach initially identified 547 ribosomes in the small cell and 849 ribosomes in the large cell. Fitting approximate cell boundaries in section 2.1.2 reduced these ribosome counts to 503 and 820 for the small cell and large cell, respectively.

In our second approach, tilt-series were preprocessed using Warp v1.0.9 (Tegunov and Cramer, 2019) for sub-frame motion correction and 3D-CTF estimation. Tilt-series were aligned using IMOD and the final reconstructions were created in Warp for subsequent processing. Template matching of ribosomes within tomograms was performed in Warp, using an initial ribosome template generated from about 200 manually picked particles from Syn3A tomograms using IMOD to avoid template bias. Extracellular particles were initially discarded based on cell boundaries defined in Dynamo. Obvious false positives (e.g., membrane segments, ice particles) were manually removed. The remaining particles were used for 3D alignment and classification in Relion v3.1 (Scheres, 2012). For each cell of interest, particles were subject to successive rounds of binary classification with a large (500 Å or 83 binned pixels) mask, the class which contained particles that did not appear as ribosomes were removed from subsequent rounds. This was done until the two classes reached about equal population. A schematic of the overall process is presented in Supplementary Figure S3. Coordinates and orientations of the remaining particles were imported into Amira for visualization. While starting with a lower correlation threshold, this second approach resulted in 718 ribosomes in the small cell and 1,136 ribosomes in the large cell, as the quality of fit increased. An additonal round of binary classification, deemed too restrictive, gave counts similar to the first approach. Fitting approximate cell boundaries in section 2.1.2 reduced these ribosome counts to 684 and 1,095 for the small cell and large cell, respectively.

**FIGURE 2:**
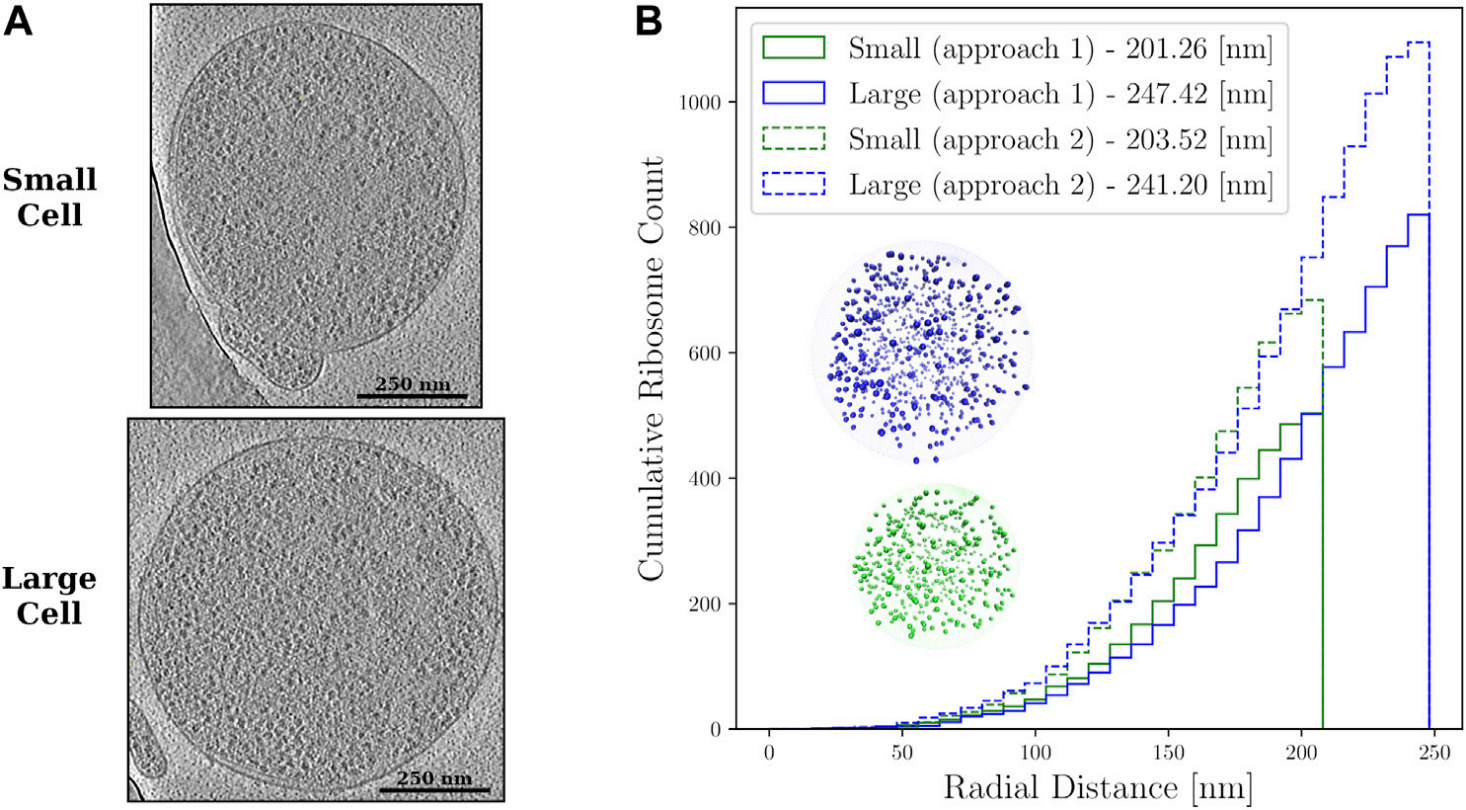
**(A)**—Z-slices from the cryo-ET data of the small and large Syn3A cells. The ribosomes are the objects with higher density than the surrounding cytoplasm that are distributed throughout the cells. **(B)**—Cumulative ribosome distributions with 8 nm bins in the reconstructed spherical geometries of a small cell of radius 201.26 nm with 503 ribosomes and large cell of radius 247.42 nm with 820 ribosomes when the first approach was used, and a small cell of radius 203.52 nm with 684 ribosomes and large cell of radius 241.20 nm with 1,095 ribosomes when the second approach was used. Also shown are the reconstructed spherical geometries resulting from the first approach to template matching.

The second approach that starts with a lower correlation threshold and includes subsequent iterative 3-D classification, is more accurate to find the final true-positive ribosomes and ribosome distributions (Lasker et al., 2021). However, it requires considerably more resources and expertise. Thus, we introduce both approaches. Even though they give slightly different distributions, both are in agreement with estimates from other experimental and computational data (Breuer et al., 2019), and notably do not significantly affect the outcome of the chromosome geometries generated, as shown in Figure 2. A summary of the ribosome counts for both approaches at each stage of our workflow are presented in Table 1.

**TABLE 1:** Summary of the ribosome distributions and cell geometries resulting from the two template matching methods for both the small and the large cell.

	Small cell		Large cell	
	Approach 1	Approach 2	Approach 1	Approach 2
Ribosomes from template matching	547	718	849	1,136
Extraneous ribosomes	44	34	29	41
Remaining ribosomes	503	684	820	1,095
SA-equivalent sphere radius (nm)	201.26	203.52	247.42	241.20
Ribosome density (ribosomes/μm^3^)	14,730	19,370	12,920	18,630

#### 2.1.2 Determining the Spherical Cell Size and Ribosome Distribution

Given a set of ribosome coordinates, the bounding membrane and shape of the deformed cell can be approximated using an ellipsoid. This was done by calculating an ellipsoid with minimal surface area that encloses the centers of all the ribosomes. The solution for the minimal surface area enclosing ellipsoid (MSAEE) was found using the minimize routine in the SciPy package with the sequential least-squares programming (SLSQP) method. To optimize the calculation, only the convex polytope of the ribosome coordinates was used to constrain the enclosing ellipsoid. The optimal enclosing ellipsoid is always constrained by four ribosomes that form a tetrahedral shape bounding the ellipsoid.

Some ribosomes identified by template matching are extraneous ribosomes, e.g., ribosomes that are present in the cell periphery but correspond to a nearby lysed cell. The extraneous ribosomes were iteratively removed from the set of coordinates and a series of ellipsoids were iteratively fit after each extraneous ribosome removal until the relative change in the ellipsoid surface area between iterations fell below 0.001%. At the end of each iteration, we choose the ribosome among the four bounding ribosomes having the greatest projection along the major axis of the enclosing ellipsoid as the extraneous ribosome and remove it. The number of extraneous ribosomes removed are summarized in Table 1 for all cases. Figure 2A shows z-slices of the cryo-ET data for both the small and large Syn3A cells. Notably, the spatial distribution of ribosomes in these cells are largely homogeneous, but small regions of about 150 nm appear to have fewer ribosomes than the surrounding cytoplasm.

After an ellipsoid approximating the membrane surface was calculated, both the ellipsoid and the enclosed ribosome coordinates were transformed to a spherical cell with equivalent surface area. A surface-area preserving transformation was chosen as previous measurements on bilayer vesicles indicated that the membrane area can only strain by approximately 5% before lysing (Needham and Nunn, 1990), thus we assume that there is a small change in volume during the blotting procedure due to mass transport of water across the membrane. The equation of an ellipsoid centered at c is $\left\{x\in {\mathbb{R}}^{3}|{\left(x-c\right)}^{T}A^{T}A\left(x-c\right)\leq 1\right\}$, where A is the matrix describing the shape of the ellipsoid, and the equation of a sphere centered at the origin is $\left\{x\in {\mathbb{R}}^{3}|x^{T}R^{-2}x\leq 1\right\}$, where R=RI and *R* is the radius of the sphere. For ribosome coordinates, $\left\{r_{i}\right\}$, the transformed coordinates, $\left\{\rho_{i}\right\}$, are given by transforming all of the coordinates to a unit sphere centered at the origin by translating them by the vector c and transforming them with the matrix A. The coordinates in the unit sphere representation are then scaled by the matrix R to a sphere with surface area equivalent to the MSAEE. The overall transformation is given by

$$\rho_{i}=\mathrm{RA}\left(r_{i}-c\right).$$ (1)

The transformation preserves the relative distances amongst the ribosomes and the shapes of the voids between the ribosomes. In a final step, the ribosome coordinates are expanded anisotropically along the semiaxes of the ellipsoid to ensure the ribosomes at the extremes reach the membrane. Two representative spherical geometries resulting from this transformation and the radial distribution of ribosomes are shown in Figure 2B.

After the transformed ribosome coordinates are determined for the spherical cell, the coordinates are projected onto the 8 nm cubic lattice used for Lattice Microbes simulations and converted to a star shape comprised of seven 8 nm cubic sites to approximate the ribosome diameter of 20 nm. The set of ribosome coordinates on the 8 nm lattice and the boundary imposed by the cell membrane then serve as constraints when generating the ensemble of chromosome configurations.

### 2.2 Modeling Bacterial Chromosome Configurations

The three primary objectives of creating a chromosome model for Syn3A are creating realistic spatial heterogeneities due to DNA crowding that are discernable at the 8 nm resolution used in spatially-resolved kinetic models of Syn3A, matching the cell architecture dictated by the cell boundary and ribosome distribution, and reproducing the intra-chromosomal interactions in chromosome conformation capture experiments through DNA-looping.

Computational models for chromosomes can be broadly classified into two groups, direct models and inverse models (Rosa and Zimmer, 2014). This distinction is not entirely black and white and it is discussed in the following paragraphs. Direct models use a minimal set of assumptions about the underlying physics of DNA or chromatin to create a polymer model, and the results of simulating the model can then be compared to experimental data (Rosa and Zimmer, 2014). These models range on length-scale from 1 bp per monomer models of the *E. coli* chromosome (Hacker et al., 2017) to 500–50,000 bp per monomer models of human chromosomes (Di Pierro et al., 2016). The models at the smallest length scales often use a Kratky-Porod model (Kratky and Porod, 1949) or a worm-like chain model for the polymer, where the persistence length of the DNA is explicitly incorporated. In contrast, the models at the largest length scales often use a Rouse model (Rouse, 1953) for the polymer, in which the monomers are assumed to be uncorrelated equilibrium globules of DNA. These models based on Rouse dynamics are well-suited for eukaryotic chromosomes on the order of $10^{7}$-$10^{8}$ bp, where the DNA is organized in nucleosomes comprised of histone octamers and other higher-order structures. A comprehensive discussion of possible interactions in the direct models of DNA polymers can be found in the review by Haddad et al. (Haddad et al. 2017) and the Minimal Chromatin Model of Di Pierro et al. (Di Pierro et al. 2016). The complexity of interactions in polymer models of DNA can range from those in homopolymer models to block copolymer models, and finally heteropolymer models (Haddad et al., 2017). Additionally, direct chromosome models can include the influence of NAPs, SMC, or bridging proteins in strings and binders models (Annunziatella et al., 2018; Ryu et al., 2021), where other particles diffuse amongst the chromosome and cause multi-point intrachromosomal interactions. After a polymer model has been specified and the chromosome of interest has been mapped to the model, molecular dynamics or Monte Carlo methods are used to sample configurations of the direct models.

Inverse models are data-driven and use large sets of experimental data to create a compatible model (Rosa and Zimmer, 2014; Oluwadare et al., 2019). The most common form of experimental data used in inverse models are chromosome contact maps resulting from 3C methods. The interaction frequencies in the contact maps are inverted to produce distance-based restraints for the chromosome models (Rosa and Zimmer, 2014). In addition to these distance-based restraints, constraints that are based on the known properties of the chromosome, such as the topology and excluded-volume effects, can be incorporated into the inverse models. A single ideal chromosome configuration that simultaneously satisfies all restraints and constraints can then be determined using iterative methods (Duan et al., 2010; Lesne et al., 2014; Hua and Ma, 2019). However, in reality, no single chromosome configuration will capture all of the interactions present in the contact map, as the contact map is an average over a population of cells. Instead, methods such as simulated annealing are used to find families of optimal chromosome configurations (Rosa and Zimmer, 2014; Junier et al., 2015). The chromosome of *M. pneumoniae* (Trussart et al., 2017) and that of *C. crescentus* (Umbarger et al., 2011) were modeled in this fashion using the Integrative Modeling Platform (Russel et al., 2012). Inverse models have also been built using maximum entropy techniques (Di Pierro et al., 2017; Messelink et al., 2021).

At the start of this study there was no experimental chromosome contact data for Syn3A, so we chose to create a direct model of the chromosome and because we intend to incorporate the chromosome configurations in simulations of whole-cell models using a lattice-based methodology (Roberts et al., 2013), we decided to use a lattice polymer model. There is a rich history of proteins and other polymers being modeled using discrete lattice models (Verdier and Stockmayer, 1962; Heilmann and Rotne, 1982; Lau and Dill, 1989; Madras et al., 1990; Dill et al., 1995). Bacterial chromosome configurations have previously been directly modeled using lattice models (Buenemann and Lenz, 2010; Messelink et al., 2021) and continuous models have been constructed by interpolating between lattice models and relaxing the system (Goodsell et al., 2018). However, none of the models satisfied all three of our requirements of 1) being at the spatial resolution needed to introduce spatial heterogeneities on the 8 nm lattice, 2) self-avoidance, and 3) able to be constrained by the cell boundary and ribosomes. We investigated modifying an existing model, such as Goodsell et al.‘s (Goodsell et al., 2018), but found that none were easily extensible.

#### 2.2.1 Growing a Self-Avoiding Polygon Model of Syn3A’s Chromosome

We model the circular chromosome of Syn3A as a circular lattice polymer. To account for the volume-exclusion effects, the circular lattice polymer is required to be strictly self-avoiding. These circular and self-avoiding configurations of monomers on a lattice are known as self-avoiding polygons (SAPs) and have been previously used to model *E. coli* and *C. crescentus* chromosomes (Buenemann and Lenz, 2010). The SAP model of Syn3A’s circular chromosome is defined on a 4 nm cubic lattice and each monomer is represented by a 4 nm × 4 nm × 4 nm cube. These monomers contain cylindrical segments of DNA 4 nm in length, which corresponds to approximately 11.8 bp per monomer. The 543 kbp chromosome of Syn3A is represented by 46,188 of these monomers. The total volume excluded by monomers in the chromosome is 2,956,032 nm^3^. At the two extremes, in the small cell with a radius of 201.26 nm, a single chromosome occupies nearly 9% of the cytoplasmic volume, and in the large cell with a radius of 247.42 nm, a single chromosome occupies just below 4% of the cytoplasmic volume.

Mathematically, the SAP configurations within the reconstructed cell geometries are described by the set of monomer coordinates, $\left\{r_{i}\right\}$, on the cubic lattice, that satisfy four different constraints, two SAP constraints, a circularity constraint (*g* ^circ^) and a self-avoidance constraint (*g* ^SA^), and two cell geometry constraints, a membrane constraint (*h* ^mem^) and a ribosome constraint (*h* ^ribo^). The circularity constraint requires that consecutive monomers are adjacent in the lattice, the self-avoidance constraint requires that no monomers share coordinates, the membrane constraint requires that the monomers remain within the cell, and the ribosome constraints require that the monomers do not intersect any ribosomes. The ribosomes in the 8 nm lattice representation are converted to a 4 nm lattice representation, where they are the same star shape, but now formed from fifty-six 4 nm cubes. These constraints are formulated mathematically using constraint functions that are equal to 1 when the constraints are satisfied and 0 when the constraints are not satisfied. All four of these constraints must be satisfied while growing and moving the SAP. While satisfying the constraints, the configurations are sampled from the canonical ensemble with a Hamiltonian that specifies intrachromosomal interactions, including looping, which will be referred to as restraints. The Hamiltonian is described in section 2.2.3.

#### 

**FIGURE 3:**
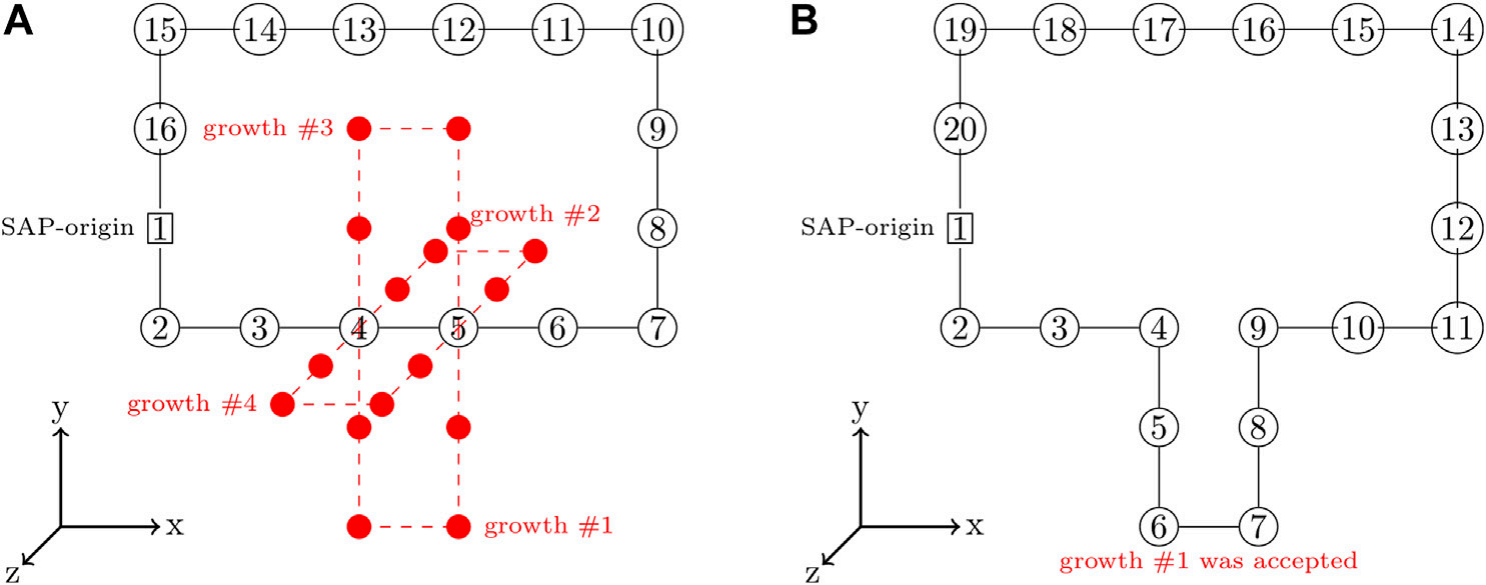
**(A)**—SAP with the set of proposed growths orthogonal to branch-point at monomers 4 and 5 shown in red. **(B)**—SAP after growth #1 with a size of 4 was accepted and incorporated into the SAP, increasing the SAP size from 16 monomers to 20 monomers.

A SAP with a greater number of monomers can be grown from an existing SAP by severing the bond between a pair of consecutive monomers and adding a closed branch orthogonal to the vector between that pair of monomers (Buenemann and Lenz, 2010; Goodsell et al., 2018). This is done in an unbiased fashion by randomly selecting consecutive pairs of monomers to serve as a branch-point and then randomly proposing growths in the orthogonal directions, an example of proposed growths is depicted in Figure 3A. Each proposed growth is only accepted if the resulting SAP satisfies all of the constraints. For example, growth #1 in Figure 3B may have been accepted because all of the other proposed growths violated the ribosome constraints. If a satisfactory growth can not be found, then the SAP is moved before searching for growths again. Pseudocode for the SAP growth algorithm is presented in Algorithm 1.

#### 2.2.2 Circularity-Preserving Moves and Proof of Ergodicity

If we start with a valid SAP configuration and then only change the configuration using moves that result in a polymer configuration still satisfying the circularity constraint, then, provided that the moves are ergodic and the new configurations are self-avoiding, we can sample SAP configurations using random sequences of the circularity-preserving moves. The proof of ergodicity follows the proof outlined in (Messelink et al., 2021).

**FIGURE 4:**
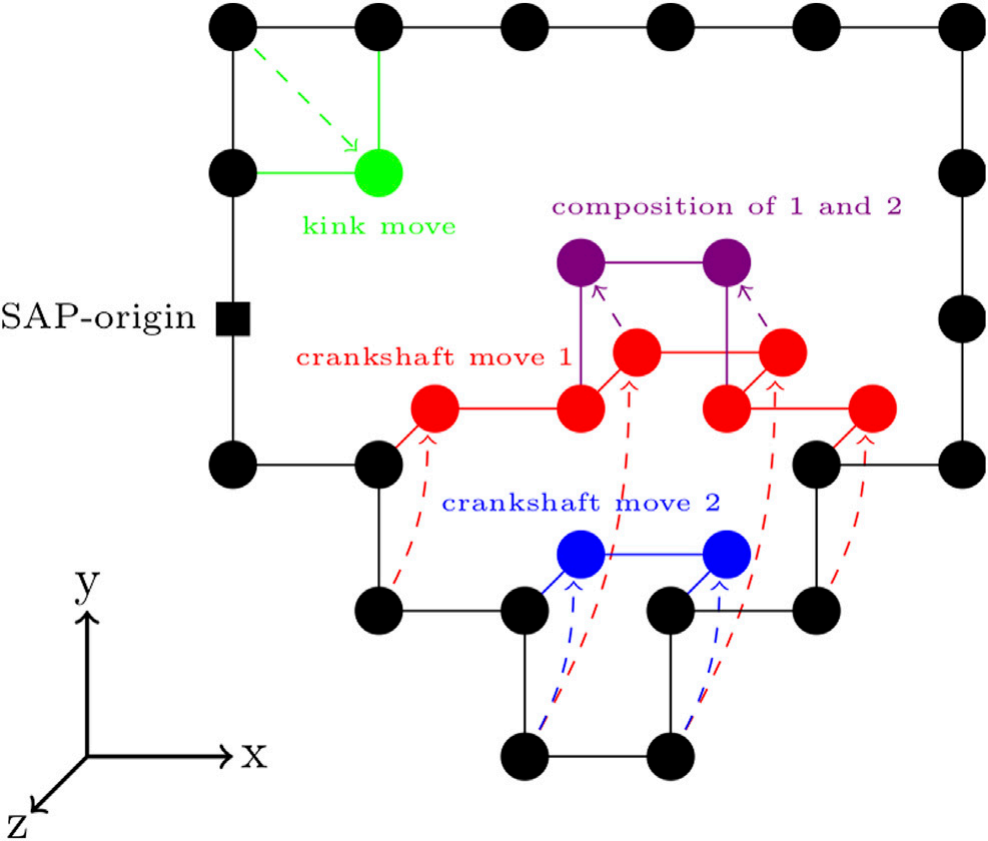
Circularity-preserving moves on a cubic lattice—An example kink move is shown in green. Two example crankshaft moves are shown in red and blue. Following a single enumeration of the set of possible crankshaft moves, multiple crankshaft moves can be made, provided that they are compatible with the crankshaft moves previously sampled from that set of possible crankshaft moves. An example of this is shown by the composition of crankshaft moves 1 and 2 in the purple.

A SAP on a lattice may be represented as a series of displacements along the cubic lattice from a starting location (Messelink et al., 2021). Displacements in the positive and negative Cartesian directions are denoted by $X^{+}$, $Y^{+}$, and ${\mathbb{Z}}^{+}$and $X^{-}$, $Y^{-}$, and ${\mathbb{Z}}^{-}$, respectively. To ensure circularity, the number of positive and negative displacements should be equal for every direction on the lattice, or symbolically, $N_{A^{+}}=N_{A^{-}}$, where A=X,Y, or ℤ (Messelink et al., 2021). Traveling counter-clockwise from the origin, the SAP in Figure 4 is described by the sequence - $\left(origin\right)Y^{-}X^{+}Y^{-}X^{+}Y^{-}X^{+}Y^{+}X^{+}Y^{+}X^{+}Y^{+}Y^{+}Y^{+}X^{-}X^{-}X^{-}X^{-}X^{-}Y^{-}Y^{-}\to \left(origin\right)$.

There are a variety of circularity-preserving moves that can transform the sequence while maintaining the circularity. For our program, we chose an extension of the Verdier-Stockmeyer moveset (Verdier and Stockmayer, 1962; Sokal, 1995) with kink moves and 2 to N/2 monomer crankshaft moves. A kink move is the interchange of two symbols in a subsequence AB→BA (Messelink et al., 2021). The move labeled kink move in Figure 4 is equivalent to $X^{-}Y^{-}\to Y^{-}X^{-}$. A crankshaft move alters a motif of a specific type. The motif is a subsequence where the monomers at the start and end of the subsequence share two Cartesian coordinates (Messelink et al., 2021). Symbolically, within such a subsequence, $N_{A^{+}}\neq N_{A^{-}}$, while $N_{B^{+}}=N_{B^{-}}$ and $N_{{\mathbb{C}}^{+}}=N_{{\mathbb{C}}^{-}}$. The crankshaft move is then a rotation of magnitude π/2, π, or 3π/2 about the vector separating the monomers at the start and end of the subsequence, applied to all of the monomers between those two. Generally, the transformation of symbols within the subsequence undergoing a crankshaft move will be $A^{\pm }\to A^{\pm }$, while $B^{\pm }\to \left({\mathbb{C}}^{\pm },B^{\mp },{\mathbb{C}}^{\mp }\right)$ and ${\mathbb{C}}^{\pm }\to \left(B^{\pm },{\mathbb{C}}^{\mp },B^{\mp }\right)$. The move labeled crankshaft move 1 in Figure 4 is equivalent to $Y^{-}X^{+}Y^{-}X^{+}Y^{+}X^{+}Y^{+}\to {\mathbb{Z}}^{-}X^{+}{\mathbb{Z}}^{-}X^{+}{\mathbb{Z}}^{+}X^{+}{\mathbb{Z}}^{+}$.

**Algorithm 1:** SAP-Growth Algorithm.

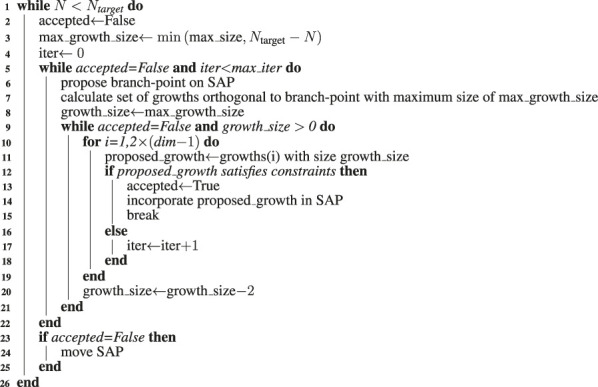

**Algorithm 2:** SAP-Move Algorithm.

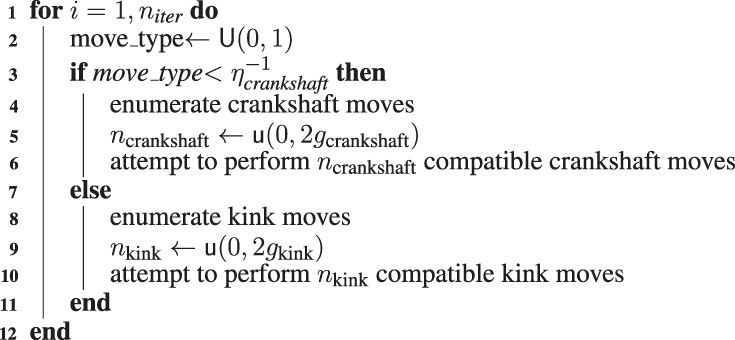

 Starting from a sequence of at least two symbol types satisifying the condition $N_{A^{+}}=N_{A^{-}}$, where A=X,Y, or ℤ, combining the kink and crankshaft moves can produce any sequence of symbols that also satisfies the condition (Messelink et al., 2021). This result allows for ergodic sampling of sequences, which is equivalent to ergodic sampling of polymer configurations satisfying the circularity constraint. However, the Verdier-Stockmeyer moveset is known to be non-ergodic for self-avoiding walks (SAWs) and SAPs due to the presence of knotted configurations (Madras and Sokal, 1987; Madras et al., 1990) and there is the additional challenge of confinement imposed by the ribosome and the cell boundary constraints. We attempted to mitigate these issues by incorporating the extended crankshaft moves and growing the SAPs to sample configurations that would otherwise be inaccessible by a single SAP being dynamically sampled using a Markov chain Monte Carlo method.

The relative frequencies of the kink moves and crankshaft moves have significant impact on the overall speed of the algorithm and are linked to the ergodicity (Sokal, 1995). The speed of the algorithm can be improved by performing multiple kink or crankshaft moves from a single enumeration of all possible kink or crankshaft moves in the current configuration, respectively. However, following the single enumeration, in addition to satisfying the SAP and spatial constraints, all kink or crankshaft moves performed must be compatible.

The list of possible kink moves are stored as an array of three element vectors of monomer indices, (i−1,i,i+1), where the *i*-th monomer in the middle will be moved by interchanging two of its coordinates that match with the coordinates of the i−1-th and i+1-th monomers. After at least one kink move is proposed and accepted, all following kink moves may not have their i−1-th or i+1-th monomers be one of the middle monomers that was moved in the previously accepted kink moves. Proposed kink moves are then rejected based on this condition. The list of possible crankshaft moves are stored as an array of two element vectors (i,j) of monomer indices, where i<j and *i* and *j* are the monomers defining the ends of the subset of the SAP which will be transformed by the crankshaft moves, and an array of two element vectors (d,ω), describing the length of the SAP subset, *d*, and the direction around the SAP in which the SAP subset is defined, ω. After at least one crankshaft move has been accepted, all following crankshaft moves must have their $\left(i^{\prime },j^{\prime }\right)$ either both belonging to the SAP subset that was moved by the crankshaft move or both not belonging to the SAP subset that was moved. Proposed crankshaft moves are then rejected based on this condition.

Crankshaft moves are the most computationally expensive to both enumerate and sample; however, they cause the fastest change in the configuration. The naive solution to this problem was to assign a frequency at which crankshaft moves were performed, $\eta_{\text{crankshaft}}$, and multiplicities for the number of kink and crankshaft moves that were performed after a single enumeration of kink or reflect moves, $g_{\text{kink}}$ and $g_{\text{crankshaft}}$, respectively. These parameters describing the sampling were then manually adjusted. Using this methodology prevents ergodic sampling from ever occurring. This can be illustrated by considering the fact that as long as crankshaft moves are sampled in batches of $g_{\text{crankshaft}}$ every $\eta_{\text{crankshaft}}$ iterations, then unless only a single crankshaft move is possible, there will never be an instance in which a kink move is sampled immediately after a crankshaft move. The inverse case is also true. An alternative is to randomly select the iterations at which crankshaft moves will be enumerated and performed, where the probability is given by $p_{\text{crankshaft}}=\eta_{\text{crankshaft}}^{-1}$. Once the move type is determined using this criteria, randomly sample the number of moves to be performed from a distribution whose mean is equal to the multiplicity of the respective move type. For example, in the case of discrete uniform distributions $n_{\text{kink}}=u\left(0,2g_{\text{kink}}\right)$ and $n_{\text{crankshaft}}=u\left(0,2g_{\text{crankshaft}}\right)$. Now there exists the possibility that any sequence of kink and reflect moves may be sampled. Pseudocode for the SAP movement algorithm is presented in Algorithm 2.

#### 2.2.3 Energy Functions and Metropolis-Hastings Sampling

The Hamiltonian for the SAP model of the chromosome has three contributions, a bending energy related to the stiffness of DNA, a nearest-neighbor interaction, and a harmonic interaction acting as a restraint to recreate the effect of DNA looping.

$$ℋ={ℋ}^{\text{bend}}+{ℋ}^{\text{n}.\text{n}.}+{ℋ}^{\text{loops}}$$ (2)

The contribution to the Hamiltonian due to the bending stiffness of linear DNA is

$${ℋ}^{\text{bend}}\left(\left\{r_{i}\right\}\right)=-\kappa \overset{N-1}{\underset{i=2}{\sum }}\left(r_{i+1}-r_{i}\right)\cdot \left(r_{i}-r_{i-1}\right)$$ (3)

and is parameterized by the bending energy per unit length squared, κ. This Hamiltonian incurs an energy penalty for every bend in the lattice polymer and can be used to model the stiffness of a polymer, a quantity often characterized by the persistence length. One interpretation of the persistence length, $l_{p}$, is the constant describing the exponential rate at which the polymer orientations become decorrelated (Brinkers et al., 2009; Hsu and Binder, 2012; Zhang et al., 2019)

$$\frac{{\langle \left(r_{i+s+1}-r_{i+s}\right)\cdot \left(r_{i}-r_{i-1}\right)\rangle }_{\text{mono}.}}{l^{2}}=\text{exp}\left(-sl/l_{p}\right),$$ (4)

where ${\langle f\left(r_{i}\right)\rangle }_{\text{mono}}$ is the average over the *N* monomers in the configuration and *l* is the lattice size. Consider the case of a SAW on a cubic lattice, in which the lattice polymer can become immediately decorrelated, thus consider the case when s=1

$${\langle \left(r_{i+1}-r_{i}\right)\cdot \left(r_{i}-r_{i-1}\right)\rangle }_{\text{mono}.}=\frac{1}{N-2}\left[\overset{N-1}{\underset{i=2}{\sum }}\left(r_{i+1}-r_{i}\right)\cdot \left(r_{i}-r_{i-1}\right)\right]=-\frac{{ℋ}^{\text{bend}}\left(\left\{r_{i}\right\}\right)}{\left(N-2\right)\kappa }pt(5)$$

leading to an equation with the bending Hamiltonian parameterized by κ. Assuming the lattice polymer is in thermal equilibrium at inverse temperature $\beta =1/k_{B}T$, we can take a thermal average of this equation

$$-\frac{\langle {ℋ}^{\text{bend}}\left(\left\{r_{i}\right\}\right)\rangle }{\left(N-2\right)\kappa l^{2}}=\text{exp}\left(-l/l_{p}\right)$$ (6)

and κ can be calculated by solving this root-finding problem through Monte Carlo sampling of SAW configurations using Wang-Landau sampling (Wang and Landau, 2001). In this study, the value of *κ* *l* ^2^ (3.872*k*_*B*_ *T*) was estimated using the exact solution for a non-reversal random walk and the consensus persistence length for DNA of 50 nm (Vologodskii et al., 1992; Manning, 2006; Brinkers et al., 2009; Geggier et al., 2010; Mantelli et al., 2011).

$$\kappa =-\frac{1}{\beta l^{2}}\text{log}\left[\frac{e^{l/l_{p}}-1}{4}\right]$$ (7)

The contribution to the Hamiltonian due to pairwise nearest-neighbor interactions is

$${ℋ}^{\text{n}.\text{n}.}=\epsilon \overset{N-1}{\underset{i=1}{\sum }}\overset{N}{\underset{j=i+1}{\sum }}\delta^{K}\left(l-|r_{i}-r_{j}|\right)$$ (8)

and was used to tune the excluded-volume effects of DNA (*ϵ* = *k*_*B*_ *T*). Lastly, the contribution to the Hamiltonian when looping restraints are imposed is

$${ℋ}^{\text{loops}}\left(\left\{r_{i}\right\}\right)=\overset{N-1}{\underset{i=1}{\sum }}\overset{N}{\underset{j=i+1}{\sum }}k_{ij}|r_{i}-r_{j}{|}^{2}$$ (9)

These pairwise harmonic interactions were used to create looping between portions of chromosome bound by SMC proteins (*k*_*ij*_ *l* ^2^ = 10,000*k*_*B*_ *T*).

A Markov chain Monte Carlo algorithm (Metropolis et al., 1953; Hastings, 1970) was used to sample configurations governed by this Hamiltonian from the canonical ensemble. We use the Metropolis criterion, $A\left(\left\{{r_{i}}^{\text{′}}\right\},\left\{r_{i}\right\}\right)=\text{min}\left(1,P\left(\left\{{r_{i}}^{\text{′}}\right\}\right)/P\left(\left\{r_{i}\right\}\right)\right)$, (Metropolis et al., 1953), for the acceptance probability of moving from the current configuration, $\left\{r_{i}\right\}$, to the proposed configuration, $\left\{{r_{i}}^{\text{′}}\right\}$. The probability of a configuration satisfying the SAP constraints (*g* ^circ^ and *g* ^SA^) and geometric constraints (*h* ^mem^ and *h* ^ribo^) is

$$P\left(\left\{r_{i}\right\}\right)=\frac{1}{Z}\left[g^{\text{circ}}\left(\left\{r_{i}\right\}\right)g^{\text{SA}}\left(\left\{r_{i}\right\}\right)\right]\times \left[h^{\text{mem}}\left(\left\{r_{i}\right\},R\right)h^{\text{ribo}}\left(\left\{r_{i}\right\},\left\{\rho_{i}\right\}\right)\right]\times \text{exp}\left[-\beta ℋ\left(\left\{r_{i}\right\}\right)\right],$$ (10)

where Z is the canonical partition function of the system found by summing over all possible configurations of *N* monomers on a cubic lattice. Assuming the current configuration, $\left\{r_{i}\right\}$, and the proposed configuration, $\left\{{r_{i}}^{\text{′}}\right\}$, always satisfy the circularity constraint because they are generated from sequences of circularity-preserving moves, then the ratio of probabilities is

$$\frac{P\left(\left\{{r_{i}}^{\text{′}}\right\}\right)}{P\left(\left\{r_{i}\right\}\right)}=\frac{g^{\text{SA}}\left(\left\{{r_{i}}^{\text{′}}\right\}\right)h^{\text{mem}}\left(\left\{{r_{i}}^{\text{′}}\right\},R\right)h^{\text{ribo}}\left(\left\{{r_{i}}^{\text{′}}\right\},\left\{\rho_{i}\right\}\right)}{g^{\text{SA}}\left(\left\{r_{i}\right\}\right)h^{\text{mem}}\left(\left\{r_{i}\right\},R\right)h^{\text{ribo}}\left(\left\{r_{i}\right\},\left\{\rho_{i}\right\}\right)}\times \text{exp}\left(-\beta \left[ℋ\left(\left\{{r_{i}}^{\text{′}}\right\}\right)-ℋ\left(\left\{r_{i}\right\}\right)\right]\right).$$ (11)

Additionally, if the proposed configuration satisfies the self-avoidance and geometric constraints, which can be determined without evaluating energy changes, then the acceptance probability given by the Metropolis criterion, $A\left(\left\{{r_{i}}^{\text{′}}\right\},\left\{r_{i}\right\}\right)=\text{min}\left(1,e^{-\beta \text{Δ}E}\right)$, is simply a function of the energy difference, $\text{Δ}E=ℋ\left(\left\{{r_{i}}^{\text{′}}\right\}\right)-ℋ\left(\left\{r_{i}\right\}\right)$, and the sampling favors low-energy configurations that better agree with the stiffness of DNA, the excluded-volume effects, and the DNA-looping restraints.

#### 2.2.4 Summary of Complete Algorithm for Generating Chromosome Configurations

**FIGURE 5:**
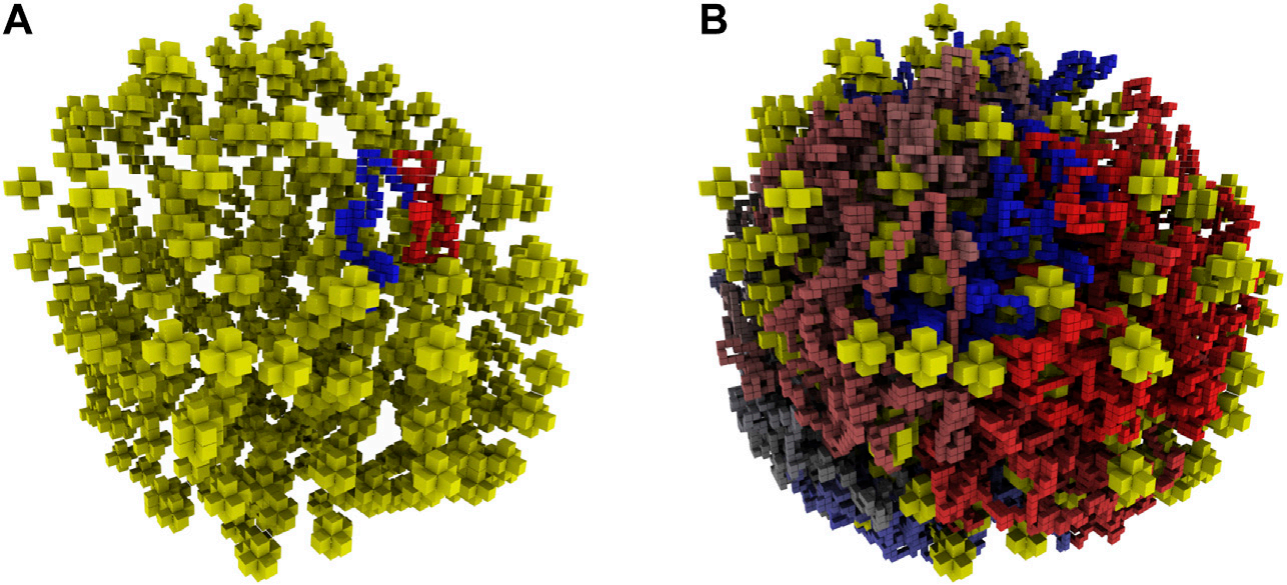
**(A)**—Reconstructed ribosome distribution in the small cell. The 100 monomers on either side of the origin are shown in red and blue. Ribosomes are depicted as yellow stars in the 8 nm lattice representation. **(B)**—Complete chromosome configuration generated on the 4 nm lattice within the reconstructed architecture of the small cell. The circular chromosome is colored starting at the origin as red to grey to blue, before returning to the origin where blue and red meet.

The final algorithm generated chromosome configurations by alternating cycles of growing and moving the SAP configurations to relax the newly grown portion. Pseudocode for the final algorithm is presented in Algorithm 3. In an early implementation, a single relaxation occurred after the growth was completed, but it was found that the alternating cycles of growth and relaxation were required because the combined effects of confinement and the exponentially increasing attrition rate due to violations of the self-avoidance constraint became overwhelming as the SAP grew larger. The relative frequencies and durations of these alternating growth and relaxation cycles were chosen empirically to maximize the speed of generating relaxed configurations of the complete chromosome. An example of how the alternating growth and relaxation cycles affect the total energy over the course of a simulation for a test case with 5,000 monomers is presented in Supplementary Figure S4. While this procedure more rapidly relaxes the system, the exponentially increasing attrition rate of rejected moves prevents us from definitively stating that we reach equilibrium in the system with 46,188 monomers. The relative frequencies and durations are described by functions, τ and σ, both are dependent on the current number of monomers, *N*, and separately depend on empirical parameter vectors, α and γ, respectively. The algorithm was implemented in Fortran 90 and a single chromosome configuration of 46,188 monomers can be generated in approximately 12–14 h on a single CPU core at 3.5 GHz. The algorithm is embarrassingly parallel and the program uses OpenMP to generate multiple configurations simultaneously. An example reconstructed cell architecture is shown in Figure 5A and the resulting constrained chromosome configuration is shown in Figure 5B.

**Algorithm 3:** Complete Algorithm For Generating Chromosome Configurations.

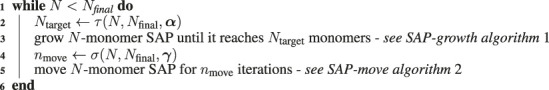

**FIGURE 6:**
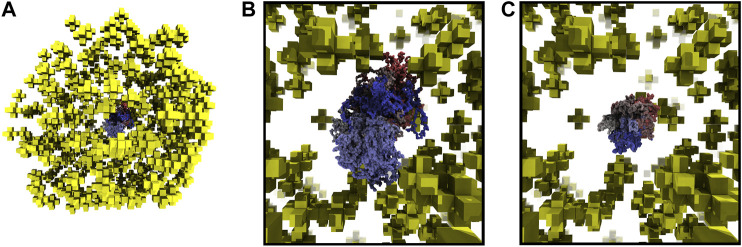
**(A)**—Centroid of 30 chromosome configurations is shown within the ribosome distribution. The same color scheme for centroid is used as for the chromosome in Figure 5. **(B)**—Magnified view of centroid in 6A calculated from 30 configurations, radius of gyration is 24.93 nm. **(C)**—Magnified view of centroid calculated from 90 configurations, radius of gyration is 11.99 nm.

Starting with fixed ribosome positions and cell orientation from the cryo-ET, we initialize the configurations by randomly placing a circular fragment of the chromosome and then independently generate hundreds of chromosomes within an otherwise identical cell. To test if the monomers along the chromosome are identically distributed within the cell, we calculate the centroid of the ensemble of chromosome configurations. The monomer coordinates of the centroid are the ensemble averages of the monomer coordinates in the chromosome configurations. The center of mass of a sphere is at its center, thus we expect the centroid of the ensemble of chromosome configurations to be approximately located at the center of the spherical cell. We find the centroid of 30 configurations to be located in the center of the cell, as shown in Figure 6A. Furthermore, if the number of identically distributed chromosome configurations is increased, we expect the centroid to collapse to the center, which we quantify with its radius of gyration. The centroid of 30 configurations in Figure 6B has a radius of gyration of 24.93 nm and it is reduced to 11.99 nm when the centroid is calculated from 90 configurations, as shown in Figure 6C.

Other bacteria that are not genetically-minimal have additional regulatory systems used to control their chromosome organization, such as attachment organelles and parAB*S* systems. Due to these regulatory systems, their chromosomes show consistent configurations that correlate the genomic position with the internal structure of the cell (Umbarger et al., 2011; Marbouty et al., 2015; Trussart et al., 2017) and this is reflected in their centroids. For example, in a model of *M. pneumoniae*’s chromosome, Trussart et al. saw a consistent alignment and interweaving of the two chromosome arms of the centroid (Trussart et al., 2017). As a comparison, we tested fixing the origin of our chromosome at the membrane and found that the centroid had a consistent alignment of the two chromosome arms at the fixed origin and monomers near the origin were found near the membrane (data not shown). Since there are no interactions correlating the genomic position and the internal structure of the cell, we compared the average radius of gyration for chromosome configurations generated in the small cell with and without ribosomes present to test the excluded volume effect of ribosomes. The average radius of gyration without ribosomes was 145.40 nm and was 133.59 nm when ribosomes were present. We also tested the effect of further increasing the number of ribosomes by randomly placing 497 ribosomes in addition to the 503 from the tomogram in the small cell and found that the average radius of gyration further decreased to 124.29 nm. We attribute this reduction in the average radius of gyration to the additional confinement caused by the volume exclusion of the ribosomes.

### 2.3 3C-Seq Library Preparation

JCVI-syn3A chromosome contact maps were prepared with 3C-Seq (Lioy and Boccard, 2018), a chromosome conformation capture technique reminiscent of Hi-C (Crémazy et al., 2018). The protocols differ in that following the restriction digestion of the fixed chromosome, restriction fragment ends are not filled-in with biotin-labelled nucleotides in 3C-Seq (Crémazy et al., 2018; Lioy and Boccard, 2018). The modification reduces the cost of chromosome conformation capture in prokaryotes since the requirement for biotin-labelled nucleotides is alleviated. 3C-Seq increases the diversity of restriction enzyme options available for library preparation from only enzymes that generate 5′-overhangs that can be filled-in by the Klenow fragment, to include enzymes that generate 3′-overhangs, and blunt-ends. Furthermore, sticky ends generated by restriction digestion are not “blunted” in 3C-Seq, increasing ligation efficiency since sticky-end ligation occurs more efficiently than blunt-end ligation. In addition, the absence of biotin at restriction fragment ends eliminates the requirement of removing biotin-labels from unligated ends, DNA purification following biotin removal, and enrichment of biotin-labelled ligation junctions, effectively, reducing the library preparation time by at least 30%.

Syn3A was cultured to stationary phase in 25 ml of SP4-KO medium in a 50 ml conical tube at 37°C. The cells were fixed with a final concentration of 1% formaldehyde (Sigma-Aldrich) at 25°C for 30 min and 4°C for a further 30 min. The reaction was quenched with 0.125 M glycine (Sigma-Aldrich) for 15 min at 4°C. The fixed cells were collected by centrifugation and washed twice with 1X HE pH 8.0 [10 mM HEPES (Sigma-Aldrich), 1 mM EDTA (Sigma-Aldrich)]. The cell pellet was flash-frozen with liquid nitrogen in a 1.5 ml low-binding microfuge tube and stored at −80^°^C until use. Fixed Syn3A cells were resuspended in 100 μl of 1X HE pH 8.0 and mechanically sheared with 0.5 mm glass beads (Sigma-Aldrich) using a vortex mixer. Membranous structures in the lysate were solubilised with 0.5% SDS (Sigma-Aldrich) for 15 min at 37^°^C in a Thermomixer® (Eppendorf) with shaking at 1,000 rpm. SDS was quenched with 1% Triton X-100 (Sigma-Aldrich) in 1X CutSmart buffer (NEB) for 15 min at 37^°^C in a Thermomixer® (Eppendorf) with shaking at 1,000 rpm. The extracted chromatin was digested with 100 U of NlaIII (NEB) for 3 h at 37^°^C. The reaction was terminated with 0.5% SDS (Sigma-Aldrich) for 20 min at 37^°^C. The digested chromatin was centrifuged at 20,000 xg for 1 h at 4^°^C. The supernatant was removed and the gel-like pellet was dissolved in 200 μl of nuclease-free water (ThermoFisher Scientific). The DNA concentration of the dissolved chromatin was determined using the Qubit® HS dsDNA assay kit (ThermoFisher Scientific) and the Qubit® fluorometer (ThermoFisher Scientific). 3 μg of DNA was used for ligation in 1X T4 DNA ligase buffer (NEB) supplemented with 100 μg/ml BSA (NEB) in a final volume of 1,000 μl. The reaction was carried out with 4000 CEU of T4 DNA ligase (NEB) at 16^°^C for 16 h and 25^°^C for 1 h. Ligation was terminated with 10 mM EDTA pH 8.0 (usb Corporation). Ligated DNA (the 3C library) was extracted twice with 25:24:1 phenol:chloroform:isoamyl alcohol (Sigma-Aldrich) and once with chloroform (Sigma-Aldrich). The library was precipitated with 0.1 × 1.0 M NaOAc (Sigma-Aldrich) pH 8.0, 0.025 × 5 mg/ml glycogen (Invitrogen), and 2.5 × 100% ethanol (Sigma-Aldrich) at -20^°^ C overnight. Precipitated DNA was pelleted by centrifugation and the pellet washed twice with 70% ethanol (Sigma-Aldrich). The pellet was air-dried and dissolved in 50.0 μl of 10 mM Tris (Sigma-Aldrich) pH 8.0. The 3C library was purified with 3X KAPA HyperPure beads (KAPA Biosystems) and eluted in 20.0 μl of 10 mM Tris (Sigma-Aldrich) pH 8.0.3C-Seq libraries for next-generation sequencing were prepared using the KAPA HyperPlus Kit (KAPA Biosystems) according to the manufacturer’s protocol. 3C-Seq libraries were sequenced on an Illumina® platform.

## 3 Results

### 3.1 3C-Seq and *in Silico* Contact Maps

**FIGURE 7:**
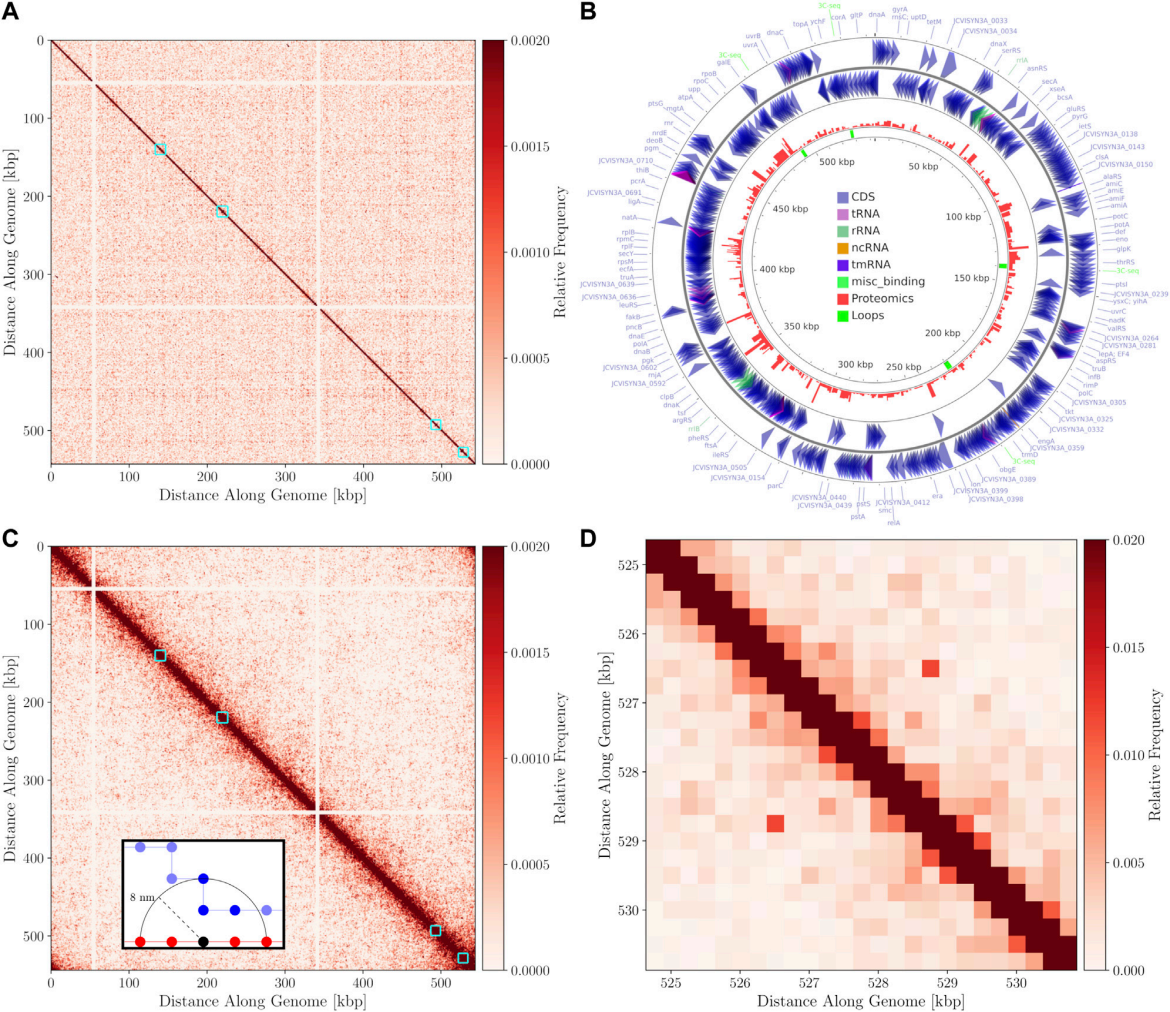
**(A)**—3C-Seq contact map at 1,000 bp resolution with the color-scale adjusted to make weak secondary features along the diagonal more apparent. The four manually annotated loops listed in Table 2 are indicated with cyan boxes. **(B)**—Circular chromosome of Syn3A with features shown as arrows and arcs around the perimeter - constructed using CGview (Petkau et al., 2010). The proteomics of a 400 nm Syn3A cell (Breuer et al., 2019) are plotted in red around the middle ring and the innermost ring contains the annotated loops in green. **(C)**—*In silico* contact map resulting from 150 configurations with looping interactions added at the positions of the manually annotated loops in Table 2. A locus size of 1,000 bp was used to match the 3C-Seq map. The interactions at the ribosomal RNA operons have been removed from the map to enhance visual clarity. Cyan squares are again used to indicate regions containing the loops. **(D)**—Magnified view of the region within the cyan square containing the fourth loop in the *in silico* contact map. The map was recalculated at a resolution of 250 bp and the maximum of the color-scale was increased to better resolve the characteristic signature of a loop.

The 3C-Seq library prepared using the restriction enzyme NlaIII had a total of 1,819,715 reads that were mapped at a resolution of 1,000 bp. A histogram of restriction digestion fragment sizes and distribution NlaIII cut sites in Syn3A’s chromosome are presented in Supplementary Figures S5,S6, respectively. The contact map was normalized to be a doubly-stochastic matrix using the matrix-balancing procedure of Knight and Ruiz (Knight and Ruiz, 2012; Rao et al., 2014) and is shown in Figure 7A. The chromosome contact map shows a primary diagonal of high interaction frequency that reflects the physical proximity of loci that lie close to each other along the primary sequence of the DNA polymer. A secondary diagonal cannot be detected implying the absence of inter-arm interactions along the chromosome. The absence of a secondary diagonal is in contrast to the chromosome contact maps of *M. pneumoniae* (Trussart et al., 2017), *B. subtilis* (Marbouty et al., 2015), and *C. crescentus* (Le et al., 2013; Tran et al., 2017). Notably, there are two regions of the chromosome that are devoid of interactions, these regions correspond to the two identical ribosomal RNA operons in Syn3A and can be seen in Figure 7B. No interactions were assigned to these regions as sequencing reads arising from either copy could not be distinguished. There are smaller secondary features along the diagonal that we interpret to be regions of high interaction due to looping. However, as this is a preliminary map with a low read depth and signal-to-noise ratio, chromosome architecture cannot be reliably interpreted and standard loop and chromosome interaction domain (CID) annotation software (Durand et al., 2016b) was unable to reliably process the map. Upon visual inspection at a resolution of 250 bp, the map shows four interactions, with distinct signatures reminiscent of loops (Fudenberg et al., 2016). Snapshots of the four interactions at 250 bp resolution in Juicebox (Durand et al., 2016a) are shown in Supplementary Figure S7. We infer the end points of these loops to be such that they fully encompass genes in the corresponding regions of the chromosome. The positions of these manually annotated loops, the genes they encompass, and the corresponding proteomics are presented in Table 2, and the loop locations within the contact map can be seen in Figure 7A.

**TABLE 2:** Loops inferred from 3C-Seq library of Syn3A. The gene annotations and locus tags are those in the NCBI entry for Syn3A’s genome (https://www.ncbi.nlm.nih.gov/nuccore/CP016816.2) and the locus tags are abbreviated to only the 4-digit number.

Start (bp)	Stop (bp)	Length (bp)	% *in silico*	Genes encompassed	Proteomics
138,324	141,557	3,233	8.7	pdhC	182
—				lpdA	182
217,523	221,263	3,740	12.7	ywjA (0371)	175
—				ywjA (0372)	65
491,413	493,784	2,371	16.7	lgt	55
—				trx	100
526,669	528,859	2,190	20.7	0877	9
—				0878	7

By comparing the annotated loops to the proteomics (Breuer et al., 2019), as shown in Figure 7B, we can investigate correlations between the relative expression levels and the locations of the loops. For reference, the average proteomics count in Syn3A is approximately 180 (Breuer et al., 2019). We will refer to the loops according to their order along the genome. The first loop encompasses the genes *pdhC* and *lpdA*, which respectively code for the E2 and E3 subunits of the PDH complex. These genes have identical proteomics counts and lower expression levels than the genes surrounding them. Upstream are genes coding for enzymes in the main pathway of the central metabolism in Syn3A (Breuer et al., 2019) and downstream are genes coding for components of the PTS system, another essential part of the central metabolism. The second loop encompasses the genes *ywjA* (0371) and *ywjA* (0372), which code for the two subunits of the flippase. This is the longest loop and the two genes within it have the greatest disparity in expression levels. The third loop encompasses the genes *lgt* and *trx*, which code for lipoprotein diacylglyceryl transferase and thioredoxin reductase, respectively. The proteomics counts of both proteins coded by these genes are lower than average, as are those of the genes immediately downstream. However, less than 10 kbp upstream is an operon for ribosomal proteins, which contains some of the most highly-expressed genes in Syn3A’s genome (Breuer et al., 2019). The fourth loop encompasses genes that code for two uncharacterized proteins, JCVISYN3A_0877 and JCVISYN3A_0878, both of which have very low proteomics counts. The expression levels of the nearby genes are similarly low. Our most consistent findings are twofold. First, the loops are all between 2 and 4 kbp in length. Second, the loops often contain genes with common expression levels.

The chromosome configurations on the 4 nm lattice, with 11.8 bp monomers, enable the calculation for contact matrices at any resolution greater than 11.8 bp per locus. Equally-sized contiguous regions of the chromosome can be classified as loci and the pairwise interactions between the loci counted according to the relative pairwise distances between monomers belonging to the loci. In the foreground of Figure 7C is a representative example of the interaction counting. When counting the total number of interactions between the red and blue loci using an arbitrary threshold distance indicated by the dashed line, the black monomer in the red loci contributes three interactions to the total interaction count between the loci. Due to a relative scarcity of chromosome models at a similar resolution in terms of bp per monomer and uncertainty about what proteins are involved in protein-DNA formaldehyde cross-linking (Dekker et al., 2002; van Berkum et al., 2010), the distance for assessing interactions can be chosen from a minimum of 4 nm corresponding to lattice spacing to a maximum of 50 nm. The maximum distance corresponds to the length of SMC proteins, which is the maximal distance spanned by a nucleoid-associated protein in Syn3A (Diebold-Durand et al., 2017; Marko et al., 2019; Ryu et al., 2021). We selected a contact radius of 8 nm because it is an integer multiple of our lattice spacing and the resulting maps show the best agreement with 3C-Seq map. This distance metric can be used alongside any locus size converted to units of monomers to generate contact matrices for ensembles of computationally generated chromosome configurations.

Contact maps were calculated for chromosome configurations within the small cell. A locus size of 1,000 bp was chosen to match the resolution of the 3C-Seq chromosome contact map. As was the case for the experimental contact map, the contact map was normalized to be a doubly-stochastic matrix using a matrix-balancing procedure. Unfortunately, the precision of the *in silico* contact maps is limited by the number of chromosome configurations used to calculate the ensemble-averaged interaction frequencies, a number many orders of magnitude lower than the number of cells in typical 3C-Seq experiments.

In the absence of a sequence-specific system, such as the parAB*S* system, dictating the global structure of the chromosome and promoting inter-arm interactions, we decided to explore a test case of introducing looping interactions at the positions of the manually annotated loops to test the efficacy of our model. We consider a loop to be successfully formed if the monomers at the endpoints are separated by less than 16 nm, the percentage of configurations with successful loop formation are shown for each loop in Table 2. One or more loops were formed in 75 of the 150 configurations. As expected, decreasing the length of the loop increased the probability that it was successfully formed. The *in silico* contact map generated from 150 configurations in the small cell is presented in Figure 7C. The contact map shows a single diagonal in Figure 7C, which is consistent with the 3C-Seq contact map and indicates that the majority of interactions are self-interactions within loci or interactions between neighboring loci. The strongest signal characteristic of a loop was observed for the fourth loop, which is the shortest, and Figure 7D shows a magnified view of the surrounding region in the contact map.

Using the 3C-Seq map, we plotted the interaction frequency as a function of genomic distance and observed a plateau after the initial decrease in interaction frequency. We fit a power law of the form $P\left(x\right)\propto x^{s}$ to two regimes within the strictly-decreasing region before the plateau, i.e. the region extending from self-interactions along the diagonal to interactions with loci at distances less than or equal to 10 kbp away, and found a range of exponents (s=−0.519 to s=−2.210). We repeated this calculation for the *in silico* map and found a narrower range of exponents than in the 3C-Seq case (s=−0.720 to s=−1.132).

Plots of the two datasets and their contact laws are presented in Supplementary Figure S8. In both cases, the steepest rate of change and largest exponent was found in the region whose lower limit corresponded to interactions of loci separated by 1 kbp. For the *in silico* case, the calculated values of *s* are in closer agreement to the value expected when confined homopolymers are organized as fractal globules (s=−1), with clearly defined territories caused by topological constraints, rather than equilibrium globules (s=−1.5) (Lua et al., 2004; Lieberman-Aiden et al., 2009; Mirny, 2011; Rosa and Zimmer, 2014; Sanborn et al., 2015). The organization of the chromosome into territories can be observed for the *in silico* case in Figure 5B as the separation into distinct colored regions.

The plateau is more pronounced in the 3C-Seq case, than the *in silico* case, and all interactions in the 3C-Seq dataset are nearly equally probable at genomic distances greater than 100 kbp. While the plateau in the 3C-Seq dataset is a characteristic of equilibrium globules (Lieberman-Aiden et al., 2009; Mirny, 2011) and some mathematically-predicted fractal globules, such as the Sierpinski triangle and inside-out Hilbert curves (Sanborn et al., 2015), we are unable to infer a topological state of the Syn3A chromosomes sampled using 3C-Seq because of the significant variations in the exponents of the power law and the sensitivity to the regime chosen for fitting. It is possible that these variations and the steep drop off are a consequence of the low coverage in the preliminary 3C-Seq map or reflect biologically relevant levels of organization.

### 3.2 Spatial Model of JCVI-syn3A

Computational modeling of spatially-resolved kinetics in Syn3A is done by simulating the reaction-diffusion master equation (RDME) in Lattice Microbes (LM) (Roberts et al., 2013; Hallock et al., 2014; Earnest et al., 2017, 2018; Bianchi et al., 2018) using a stochastic simulation algorithm. When using the RDME, physical space is discretized into a cubic lattice representation. The size of the cubic lattice dictates both the resolution of the spatial modeling and the maximum allowable timestep when modeling the kinetics, smaller lattice sizes reduce the maximum allowable timestep. A lattice size of 8 nm was chosen as an acceptable compromise between creating a high-resolution spatial model of Syn3A, while permitting simulations over biologically-relevant time scales. Each of these 8 nm lattice sites can contain a maximum of sixteen particles. Previous work on combining LM simulations and tomogram data, directly reconstructed cell architectures in LM (Earnest et al., 2017). Unfortunately, as discussed earlier, the tomograms do not show well-defined DNA strands. Instead, chromosome configurations consistent with the ribosome distributions observed in the tomograms are generated using our method, and those are used for the spatial models.

We have also used the spherical cell architecture reconstructed from the tomograms to predict the number of ribosomes involved in polysomes, the number of ribosomes at or near the membrane, and the number of ribosomes close enough to DNA to form an expressome. To predict the number of ribosomes involved in possible polysomes, we calculate the pairwise distances between all ribosome pairs in the spherical cell. Annotating any pair within a center-to-center distance of 22 nm to be in a possible polysome, as was experimentally measured in *E. coli* (Brandt et al., 2009), we calculate 194, approximately 39%, of the 503 ribosomes from the first template matching method (approach 1) are involved in possible polysomes. In the second template matching method with 3D classification (approach 2), this number increases to 373, approximately 55%, of the 684 ribosomes in possible polysomes. If we instead use a center-to-center distance of 18 nm such that the ribosomes are almost in contact, we find that 125, approximately 25%, of the 503 ribosomes from approach 1 are involved in possible polysomes. In approach 2, we find that 274, approximately 40%, of the 684 ribosomes are in possible polysomes using the 18 nm distance. In cryo-ET of a closely related organism *M. pneumoniae*, sub-tomogram averaging over many cells predicted that an average of 16.4% of ribosomes are involved in polysomes (O’Reilly et al., 2020). The discrepancies arise from several factors: First, *M. pneumoniae* only has 300 ribosomes per cell (Seybert et al., 2006; Yus et al., 2009; O’Reilly et al., 2020) and has a larger volume than Syn3A (Kühner et al., 2009), so it is not unreasonable that the higher ribosome density in Syn3A results in a larger fraction ribosomes involved in polysomes. Second, the method to define polysomes in (O’Reilly et al., 2020) exclusively looks at ribosomes that are in very close proximity and oriented so that the mRNA exit channel of one ribosome aligns with the mRNA entry of the next. In cells, polysomes are likely to be more relaxed than this configuration. Our predicted fractions of ribosomes involved in polysomes are all lower than the 70% observed in fast-growing *E. coli* using absorption spectroscopy (Phillips et al., 1969; Forchhammer and Lindahl, 1971).

To estimate the number of ribosomes on or near the membrane, we calculate the number of ribosomes within a cytoplasmic shell directly inside the membrane. We annotate a ribosome as being within the cytoplasmic shell if its center is within the shell. Using a 10 nm thick shell, the approximate radius of a ribosome, we find that 53, approximately 10%, of the 503 ribosomes from approach 1 are near the membrane. We find a similar number of ribosomes within the same distance in approach 2, 60 (9%) of the 684 ribosomes. If we extend the shell to 20 nm thick, we find that 122, approximately 24%, of the 503 ribosomes in approach 1 are near the membrane. In approach 2, we found 136, or 20%, of the 684 ribosomes are within 20 nm of the membrane. The range of our calculated fractions agrees with the observed 15% of ribosomes being membrane-bound in cryo-ET of *S. melliferum* (Ortiz et al., 2006).

Expressomes are macromolecular complexes of RNA polymerases (RNAPs) and ribosomes that couple transcription and translation, they were first identified in *E. coli* (Kohler et al., 2017). In *M. pneumoniae* a maximum of 19% of ribosomes have been identified to be in an expressome complex in which NusA and NusG help to connect or direct the mRNA from production by RNAP to the ribosome (O’Reilly et al., 2020). Given the proteomics counts of RNAP, NusA, and NusG within a 400 nm cell of 187, 238, and 464 respectively (Breuer et al., 2019), the possibility of expressome complexes emerging in the whole-cell model is certainly possible. Using the 4 nm lattice representation, we searched for possible expressomes by counting chromosome monomers directly adjacent to the star-shaped ribosomes. We find that on average there are 106 of the 503 ribosomes in approach 1 with a DNA monomer directly adjacent, a fraction of approximately 21%. From ribosomes identified in approach 2, we found that 127 of the 684 ribosomes, roughly 19%, were directly adjacent to the DNA on average. This is in good agreement with the fraction of 2.8–19% of ribosomes found to be in expressomes in *M. pneumoniae* by O’Reilly et al. (O’Reilly et al., 2020).

**FIGURE 8:**
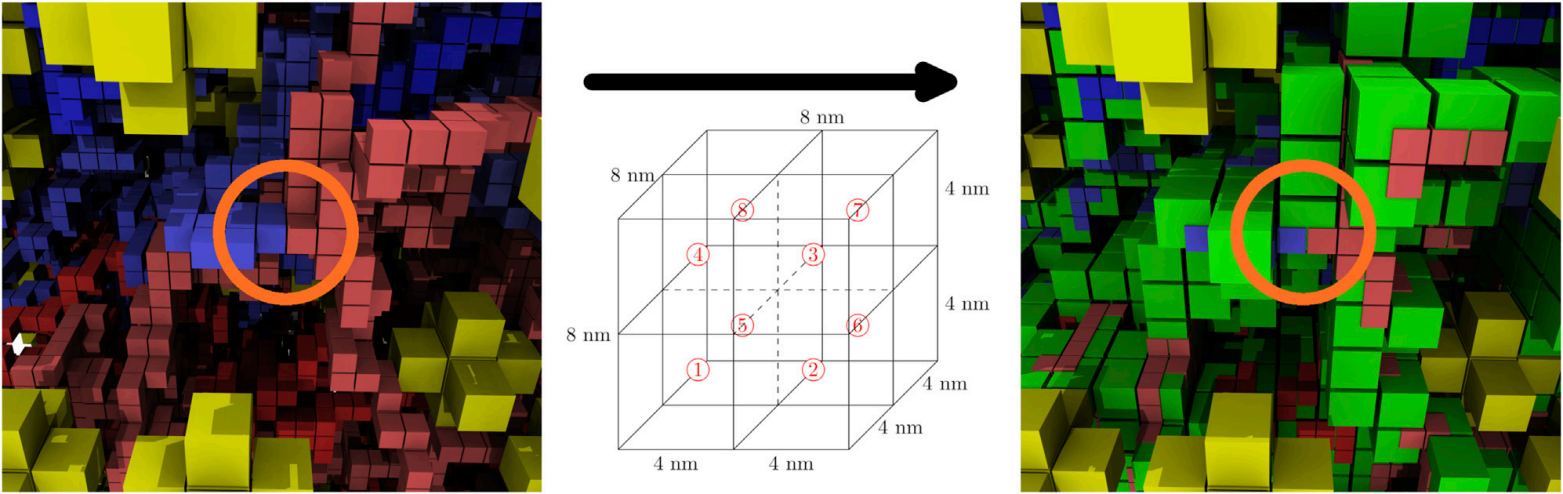
Coarse-graining, before and after—The coarse graining procedure localizes up to eight effective monomers in the 4 nm chromosome configurations (same color scheme as Figure 5) within the 8 nm chromosome lattice sites (green). Circled in orange there is an example of genomically-distant regions being localized within the same chromosome site.

The computationally-generated chromosome configurations on the 4 nm lattice are converted to the 8 nm lattice before being used in RDME simulations. The conversion is done using a coarse-graining procedure where the 4 nm effective monomers are localized within the 8 nm lattice site containing them. These 8 nm lattice sites are then identified as chromosome sites. Due to the self-avoiding nature of the chromosome model, each 8 nm chromosome site can contain up to a maximum of eight monomers, where each monomer contains 11.8 bp of DNA. The number of monomers within the 8 nm lattice site are directly converted to up to eight of the maximum of sixteen particles within a lattice site. This coarse-graining procedure preserves the overall volume exclusion of the chromosome and the spatial heterogeneities caused by varying chromosome densities throughout the cell, and allows for genomically distant pieces of the chromosome to be spatially localized within the 8 nm chromosome sites. Figure 8 shows the coarse-graining of a chromosome configuration in the small cell. The kinetic model of genetic information processing in Syn3A (Thornburg et al., 2019) can then be extended to include the effects of RNA polymerases diffusing between the spatial locations of genes within the chromosome (Weng and Xiao, 2014).

**FIGURE 9:**
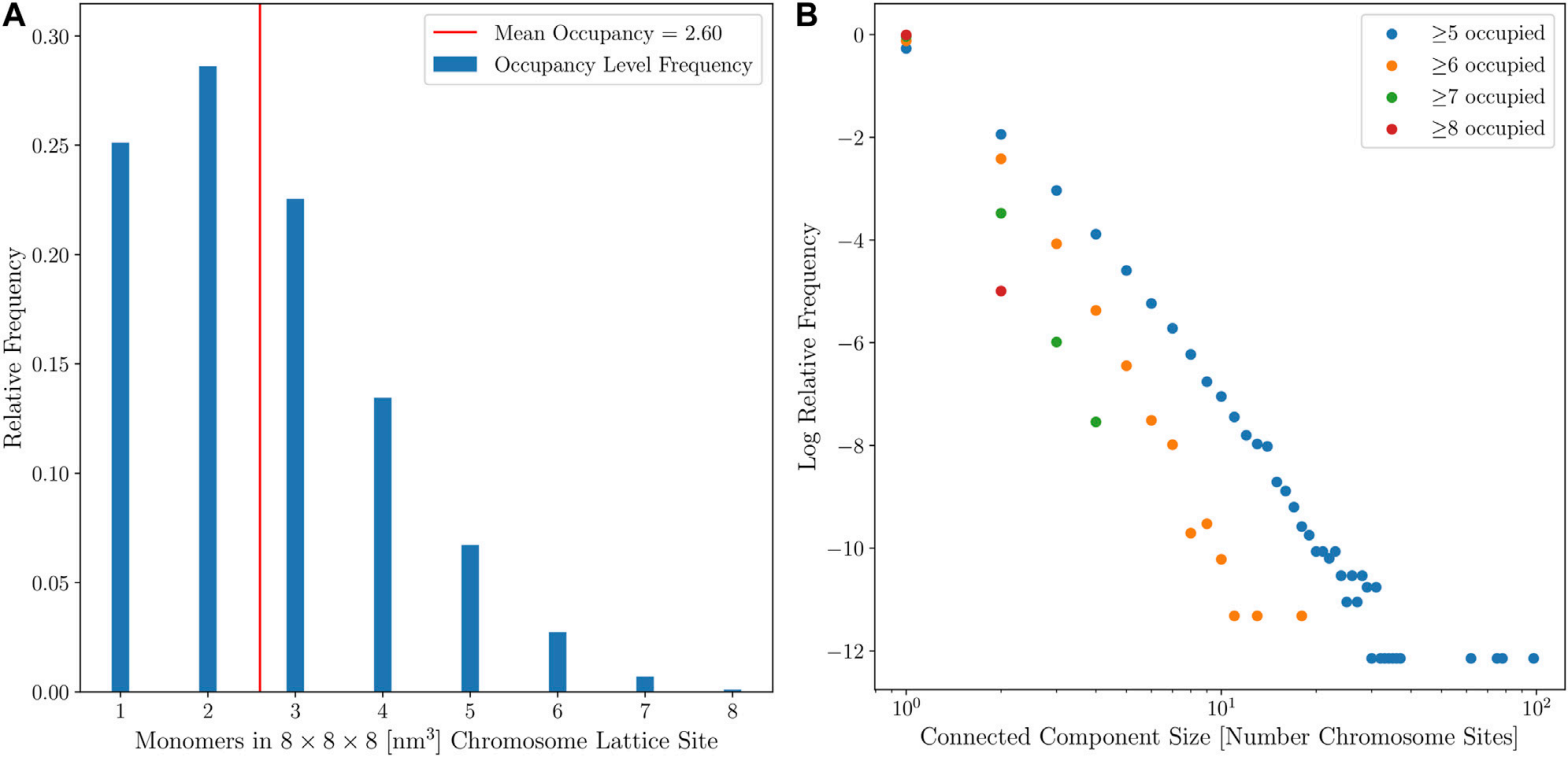
**(A)**—Relative frequencies of chromosome site monomer occupancies in the small cell from 150 configurations with no looping interactions. **(B)**—Distribution of connected-component sizes for different monomer occupancy thresholds for the small cell from 150 configurations with no looping interactions.

**FIGURE 10:**
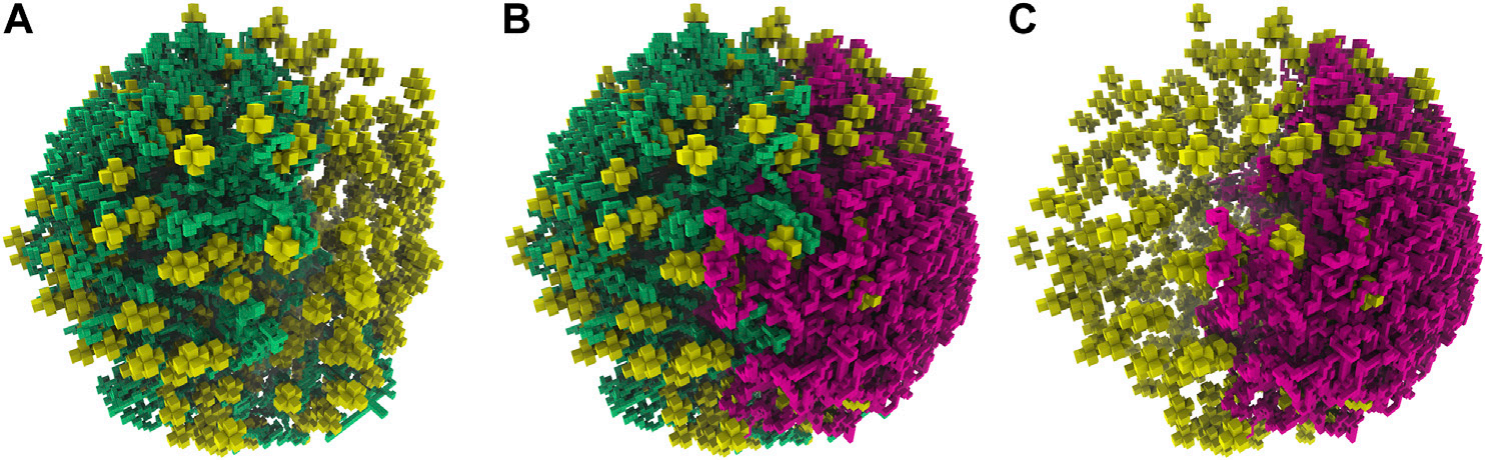
**(A)**—First chromosome (green) generated within the large cell architecture of radius 247.42 nm and containing 820 ribosomes (yellow). **(B)**—An additional second chromosome (magenta) was generated within the large cell architecture while avoiding the first chromosome (green). **(C)**—The isolated second chromosome (magenta) in the large cell architecture after the removal of the first chromosome.

Two means were used to quantify the diffusivity of the coarse-grained chromosome configurations. First, the average monomer occupancy of the coarse-grained chromosome sites was calculated, with the target average occupancy being under 3 monomers per coarse-grained chromosome site, as shown in Figure 9A. Second, connected-component labeling was used to identify contiguous regions of high monomer occupancy, where diffusing particles may become trapped for extended periods of simulation time, as shown in Figure 9B. Maintaining an acceptable number of particles per lattice site has a significant impact on the efficiency of the multi-particle diffusion used in the GPU-accelerated LM (Hallock et al., 2014). The results for the small cell when the second approach for template-matching was applied, with the high ribosome packing density, showed that all 150 computationally-generated chromosome configurations were diffuse enough to be used for RDME simulations of Syn3A. The configurations satisfy the first criterion and there were no instances in which connected-components with an occupancy greater than 8 monomers per coarse-grained chromosome site could form closed shapes on the 8 nm lattice. The large cell is presumed to be near the end of the cell cycle, after DNA replication has been completed, and a second chromosome can be placed within the cell architecture, as shown in Figure 10. We assume the small cell and large cell are representative examples of cells at the start and end of the cell cycle, respectively, and the combination of the small and large cell architectures enable whole-cell simulations of Syn3A at the start and end of the cell cycle.

## 4 Discussion

We developed a procedure to reconstruct single-cell geometries of Syn3A cells from cryo-electron tomograms. The procedure has two parts, the determination of the cell size and subsequent transformation of the ribosome distribution to a cell with spherical geometry, and the generation of circular chromosome configurations constrained by the spherical cell boundary and the ribosome distribution, and restrained by a small number of DNA loops observed in the experimental 3C-Seq map. Cell geometries were reconstructed for the small cell assumed to be at the start of the cell cycle and a large cell considered to be near the end of the cycle, when two chromosomes would be present.

The 3C-Seq chromosome contact map at a resolution of 1,000 bp has no secondary diagonal and confirms our assumption that Syn3A has no factors affecting the global structure of the chromosome. We based this assumption on our knowledge of the genome-scale gene essentiality and proteomics data, which indicated Syn3A lacked a parAB*S* system or attachment organelle. Our computational model of the chromosome reproduced this behavior while constrained by the reconstructed cell geometry. Furthermore, we generated the DNA configurations under the assumption that the DNA was in a relaxed state with limited supercoiling. This was justified due to the high abundance of proteins that modify the supercoiling state, topoisomerases and gyrases, relative to the number of RNAP, and the relatively low abundance of proteins that form topological constraints and stabilize supercoiled loops, such as HU. We were able to model local structures, whose signatures were observed in the 3C-Seq map at a resolution of 250 bp. Currently, SMC is the only annotated protein in Syn3A that can form unsupercoiled loops, so it is possible the observed loops are formed by SMC. Recent studies show that SMC functions through active loop extrusion rather than static loop stabilization (van Ruiten and Rowland, 2018), so there are potentially additional unannotated effects and/or proteins causing the experimentally observed loops or causing localization of the actively-extruding loops through preferential SMC binding at the annotated locations. Our chromosome model does not include active loop extrusion and is only capable of reproducing the results of active loop extrusion in an ensemble average sense. At this time, we wish to avoid making further definitive statements about the nature of the local structure of Syn3A’s chromosome until deeper sequencing is completed. Future experiments with additional restriction enzymes that cut the DNA at complementary positions and greater depth of the reads will help to improve our analysis.

We can speculate that the significant differences in the global chromosome organization of bacterial cells with natural genomes, such as *B. subtilis*, *C. crescentus*, and *M. pneumoniae*, to Syn3A with its synthetic genome are a result of genome minimization, both natural and targeted. The parent organism from which all variants (Syn1.0, Syn3.0, and Syn3A) are descended is *M. mycoides*, a choice that was made because *Mycoplasma* cells have small genomes that have been naturally reduced over evolutionarily-long time scales. This reduction likely occurred because they are parasitic organisms that can rely on a stable environment provided by their host. *Mycoplasmas* have dispensed of the genes that code for complex regulatory systems, such as the parAB*S* system, and the remaining genes largely code for environment-independent functions essential to all life (Hutchison et al., 2016). Chromosome organization at the local level is dictated by NAPs and the supercoiling state of the DNA. Notably, while there is a significant disparity in the relative proteomics counts of NAPs in Syn3A and naturally-occurring bacteria, with the majority of NAPs being wholly absent from Syn3A’s genome, there is no such disparity in the counts of proteins that modify the supercoiling state of the DNA. These proteins are essential to the function of Syn3A, which is not surprising due to the relationship between supercoiling and the universal process of transcription (Chong et al., 2014; Dorman, 2019).

From the reconstructed cell geometries, we estimated fractions of ribosomes that could be attached to the membrane or are complexed in possible polysomes and expressomes. We simply used distances between ribosomes and the membrane, other ribosomes, and the DNA, respectively, to predict these numbers. To confirm these estimates of the polysomes we could use the orientations of the ribosomes’ entry and exit channels such that the ribosomes can pass mRNA between each other (O’Reilly et al., 2020). The membrane-bound ribosomes can be further characterized by determining which of those ribosomes have their 50S subunit facing the membrane (Ortiz et al., 2006). Further analysis of expressomes would require a template involving the RNAP and the essential transcription factor NusA that was found to attach the RNAP to the ribosome in cryo-ET of *M. pneumoniae* (O’Reilly et al., 2020). In the same *M. pneumoniae* study, subtomogram averaging was used to more confidently assign expressome structures along with orientation of the mRNA entry site to help identify ribosomes complexed with RNAP.

The effects of ribosomes attached to the membrane or complexed in polysomes and expressomes can all be included in future whole-cell, spatially-resolved kinetic models. The configurations resulting from the SAP model of the bacterial chromosome are directly transferrable to the 8 nm lattice representation used for LM simulations of whole Syn3A cells through a coarse-graining procedure. The coarse-grained chromosome configurations specify the spatial heterogeneities caused by DNA-crowding in whole-cell kinetic models of Syn3A and define the spatial locations of genes to investigate spatial and temporal correlations in gene expression (Weng and Xiao, 2014; Thornburg et al., 2019). Future work will focus on assigning chromosomal interactions based on improved experimental 3C-Seq libraries, improving the model to include dynamic formation and relaxation of supercoiling and plectonemic loops, and incorporating dynamic representations of the chromosome (Miermans and Broedersz, 2020) within the LM simulations, which will include DNA diffusion and chromosome replication. The compactness and degree local structure of the DNA determines the accessibility of its genes to RNAP which is an important consideration in the whole-cell simulations of all the cellular networks being developed for the minimal cell JCVI-syn3A.

## Data Availability

The tilt-series in this study have been deposited in the Electron Microscopy Public Image Archive (https://www.ebi.ac.uk/pdbe/emdb/empiar/) under EMPIAR entry numbers EMPIAR-10685 (large cell) and EMPIAR-10686 (small cell). The reconstructed tomograms in this study have been deposited in the Electron Microscopy Data Bank (https://www.ebi.ac.uk/pdbe/emdb/index.html/) under EMDB entry numbers EMD-23660 (large cell) and EMD-23661 (small cell). 3C-Seq libraries of Syn3A are available from the 4TU repository (https://data.4tu.nl/) under DOI: https://doi.org/10.4121/14333618. The software used in this study can be found at https://github.com/brg4/SAP_chromosome.
