# Supplementary material for: Generating Chromosome Geometries in a Minimal Cell From Cryo-Electron Tomograms and Chromosome Conformation Capture Maps
Source: Front Mol Biosci. 2021 Jul 22;8:644133. doi: 10.3389/fmolb.2021.644133 (PMC8339304; doi:10.3389/fmolb.2021.644133)
Supplement: Supplementary file 1 [file DataSheet1.PDF]

Supplementary Material

1 SUPPLEMENTARY DATA

We have no supplementary data to include.

2 SUPPLEMENTARY TABLES AND FIGURES

2.1 Tables

We have no supplementary tables to include.

2.2 Figures

The supplementary figures are listed below.

Figure	Title
S1	Small cell cryo-ET data
S2	Large cell cryo-ET data
S3	Ribosome template matching and binary 3D classification
S4	Relaxation method comparison
S5	Restriction digestion fragment sizes
S6	Distribution of NlaIII cut sites
S7	Manually annotated loops in 3C-Seq map
S8	Contact laws for 3C-Seq and *in silico* maps

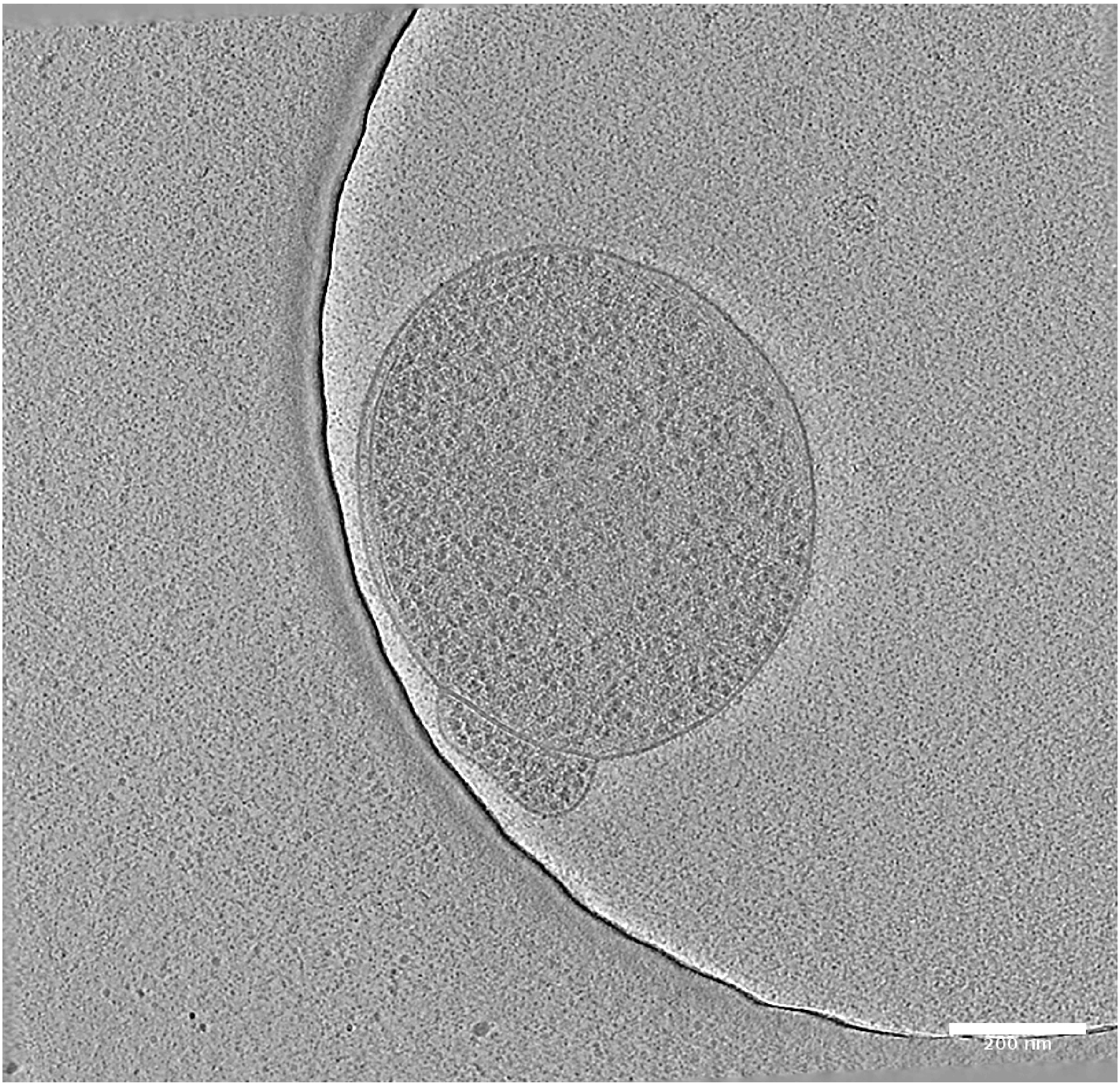

Figure S1. Small cell cryo-ET data: Z-slice of the cryo-ET data of the small cell. On the left of the cell is the edge of the hole in the grid. The ribosomes are the objects with higher density than the surrounding cytoplasm that are distributed throughout the cell.

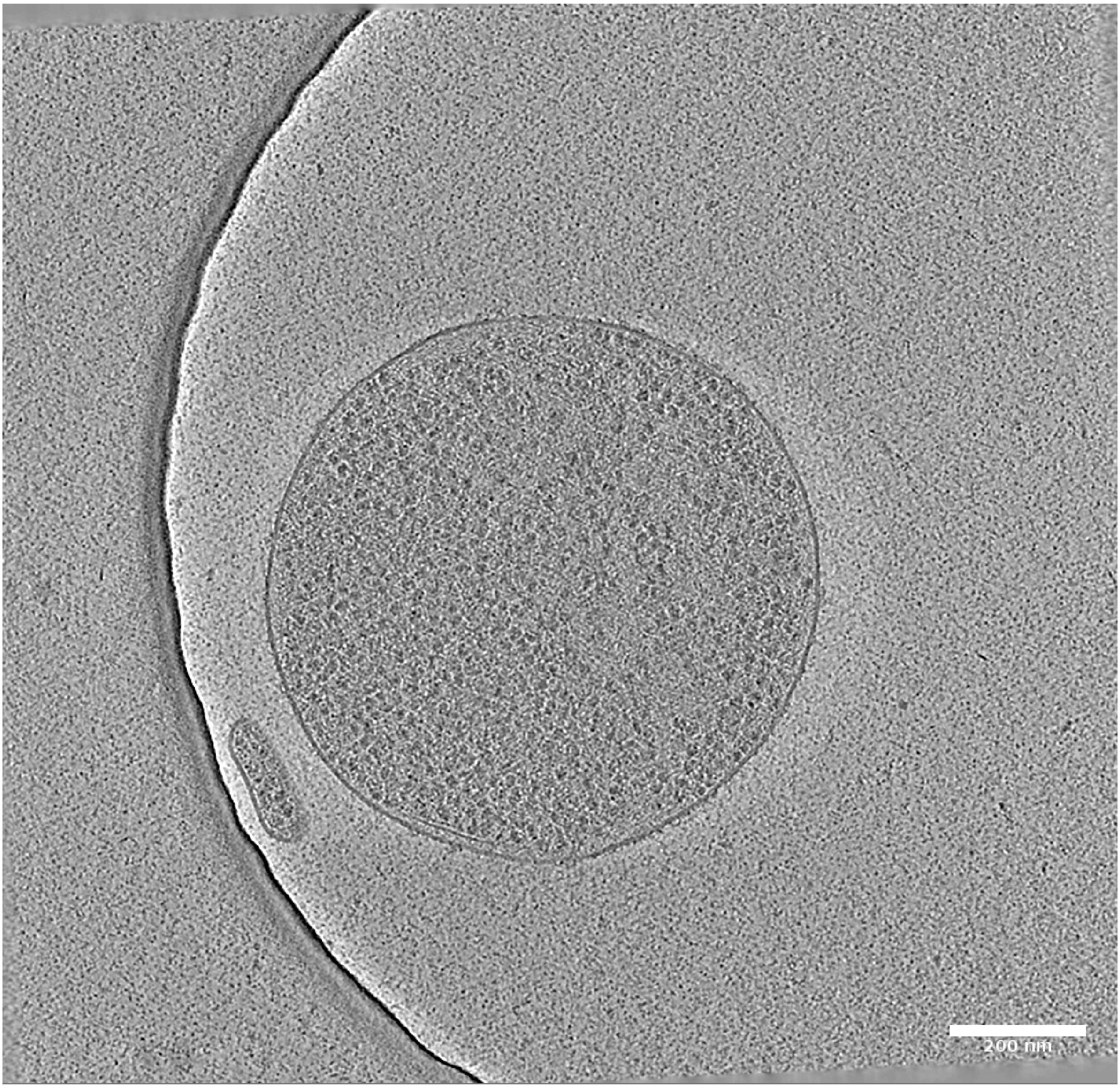

Figure S2. Large cell cryo-ET data: Z-slice of the cryo-ET data of the large cell. On the left of the cell is the edge of the hole in the grid. The ribosomes are the objects with higher density than the surrounding cytoplasm that are distributed throughout the cell.

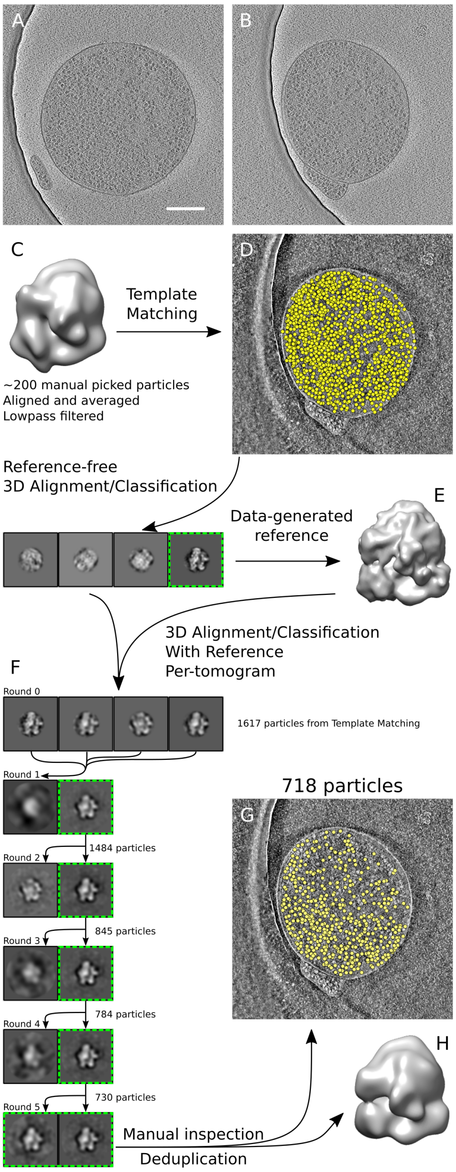

Figure S3. Ribosome template matching and binary 3D classification: A,B) Slices through tomographic reconstructions of two JCVI-Syn3A cells. Scale bar 200 nm. C) An initial average generated from 200 manually picked ribosomes was used as a reference for template matching in JCVI-Syn3A tomograms. D) Initial template matching results of B) showing matched positions as yellow dots. Template matching results were filtered with a low threshold to remove some falsepositives. Additionally, all particles outside of the cell were manually excluded. Note that the initial template matching pool still contains false positives e.g., membrane segments. E) Template matching results from all tomograms were used to create a data-generated ribosome map in Relion using reference-free 3D alignment and classification. F) Round 0: At the beginning of the iterative binary classification scheme, particles from each tomogram are aligned and classified separately, using the data-generated ribosome as a reference and a tight mask (250 Å). Rounds 1-5: Reference-free 3D classification without alignment is used to separate the particles into two classes. This is done using a large mask (500 Å). Only the class that most resembles the reference (green outline) passes to the next round of reference-free 3D classification. This continues until the two classes resemble each other, and both are kept. G) After binary classification, the results are de-duplicated and manually inspected to remove any remaining false positives e.g., membrane segments. H) Subtomogram average of the ribosomes from one single tomogram, kept at the end of the binary 3D classification.

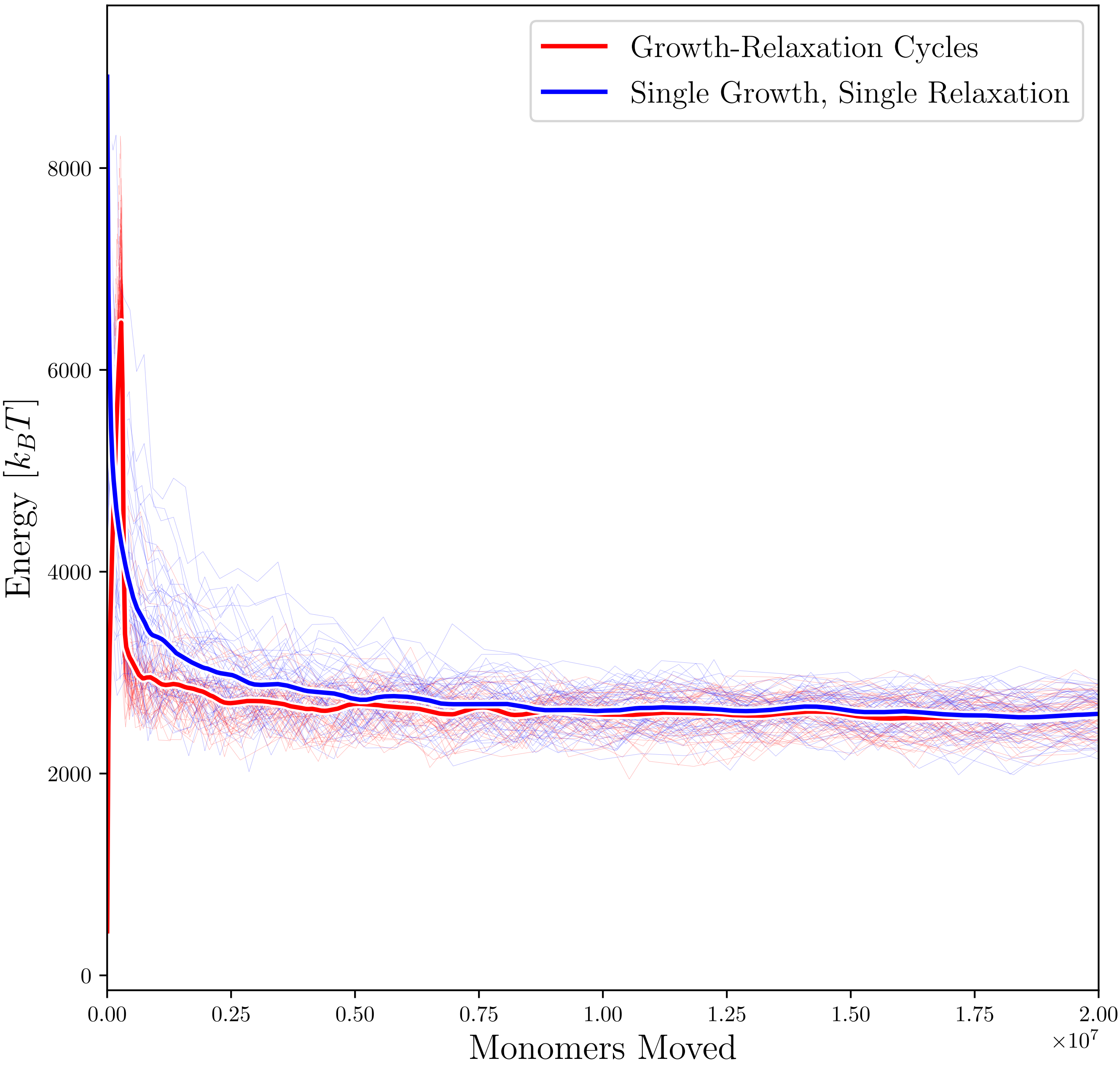

Figure S4. Relaxation method comparison: Plot comparing the total energies as a function of the number of monomers moved for 100 configurations of 5000 monomers each that were generated either using a single growth followed by a single relaxation or alternating cycles of growth and relaxation. The thick lines are the ensemble averages for each case. We use the number of monomers moved to assess the computational cost because the most computationally-expensive subroutines are those used to test the constraints for each monomer. The single growth creates a configuration with a high energy and then the system is slowly relaxed. We observe this decay in the graphs. The alternating cycles of growth and relaxation cause an increase in the average total energy until the complete extent of the configuration has been grown, which is the cause of the peak in the energy. However, the average total energy at this peak is lower than the initial average total energy of configurations resulting from a single growth. Additionally, there is a more rapid relaxation from this peak down to the equilibrium state. We see that on average, the alternating growth and relaxation cycles require fewer monomers to be moved to reach a lower energy state.

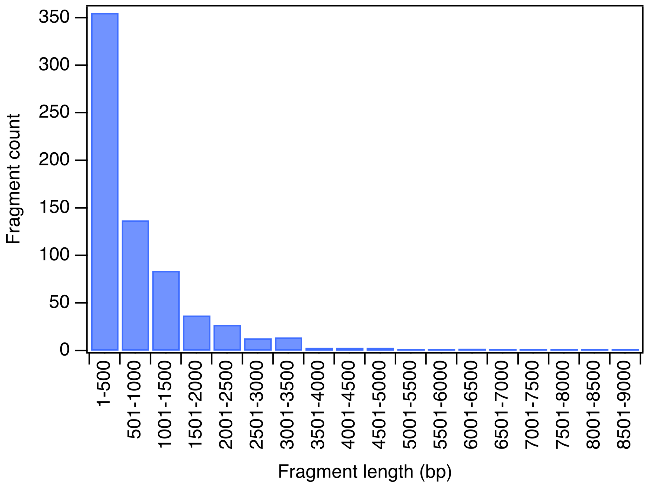

Figure S5. Restriction digestion fragment sizes: A histogram of the length of restriction digestion fragments generated by the digestion of Syn3A's chromosome with NlaIII (cut site: CATG).

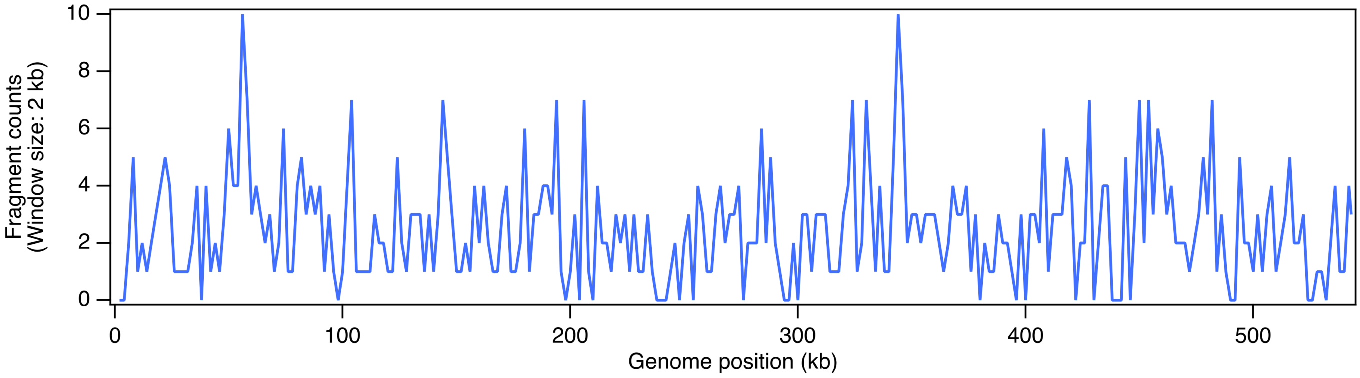

Figure S6. Distribution of NlaIII cut sites: The distribution of NlaIII (cut site: CATG) digestion sites along Syn3A's chromosome. There are 680 cut sites in Syn3A's genome which results in an average spacing of 799 bp between cut sites.

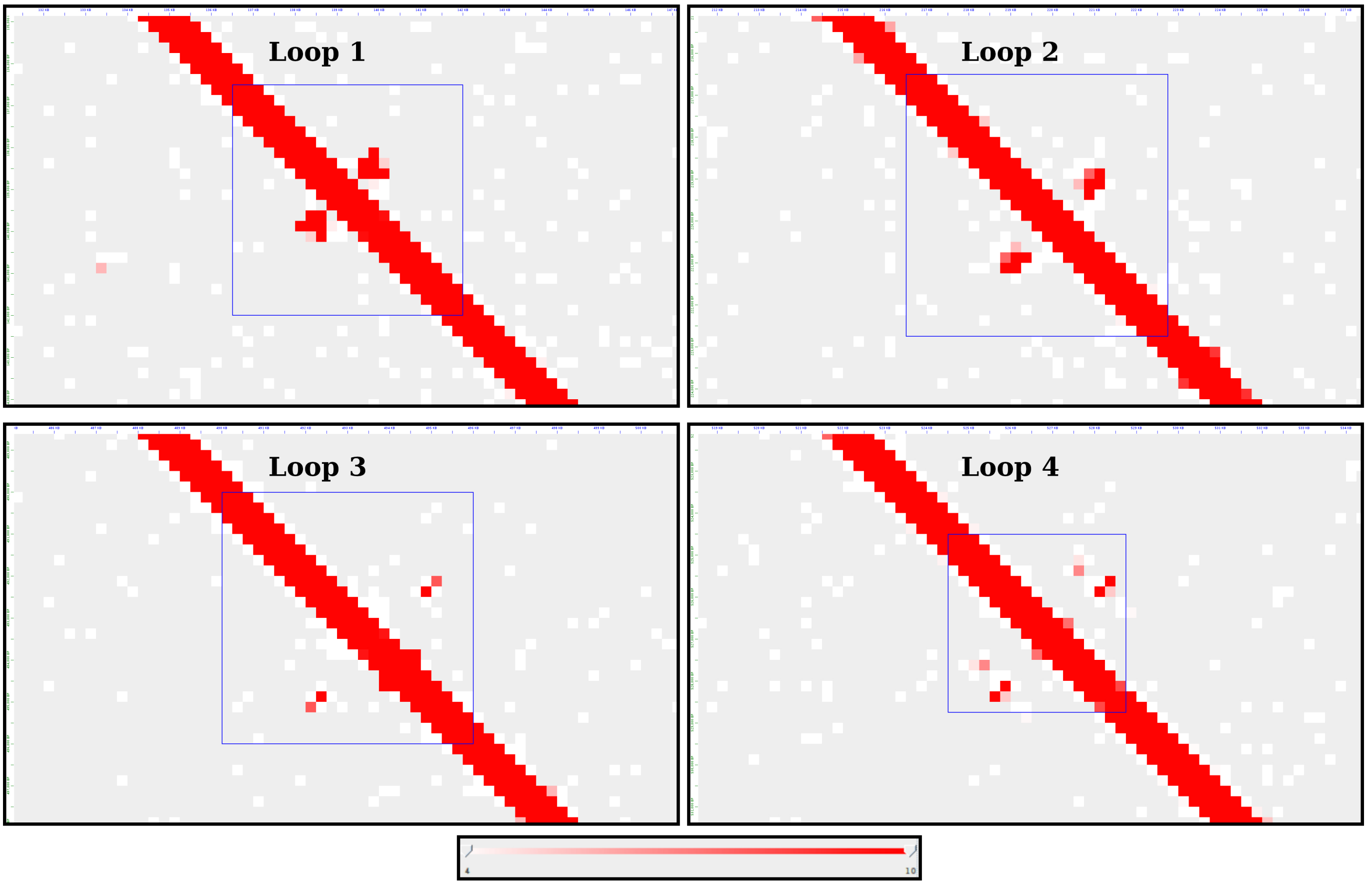

Figure S7. Manually annotated loops in 3C-Seq map: Magnified views of the four interactions manually annotated as loops in the 3C-Seq contact maps at a resolution of 250 bp - visualized using Juicebox (Durand et al., 2016). The loop locations were annotated as the following, so as to encompass complete genes in the corresponding regions: Loop 1 = 138,324-141,557 bp, Loop 2 = 217,523-221,263 bp, Loop 3 = 491,413-493,784 bp, and Loop 4 = 526,669-528,859 bp. The common color scale is displayed at the bottom of the image.

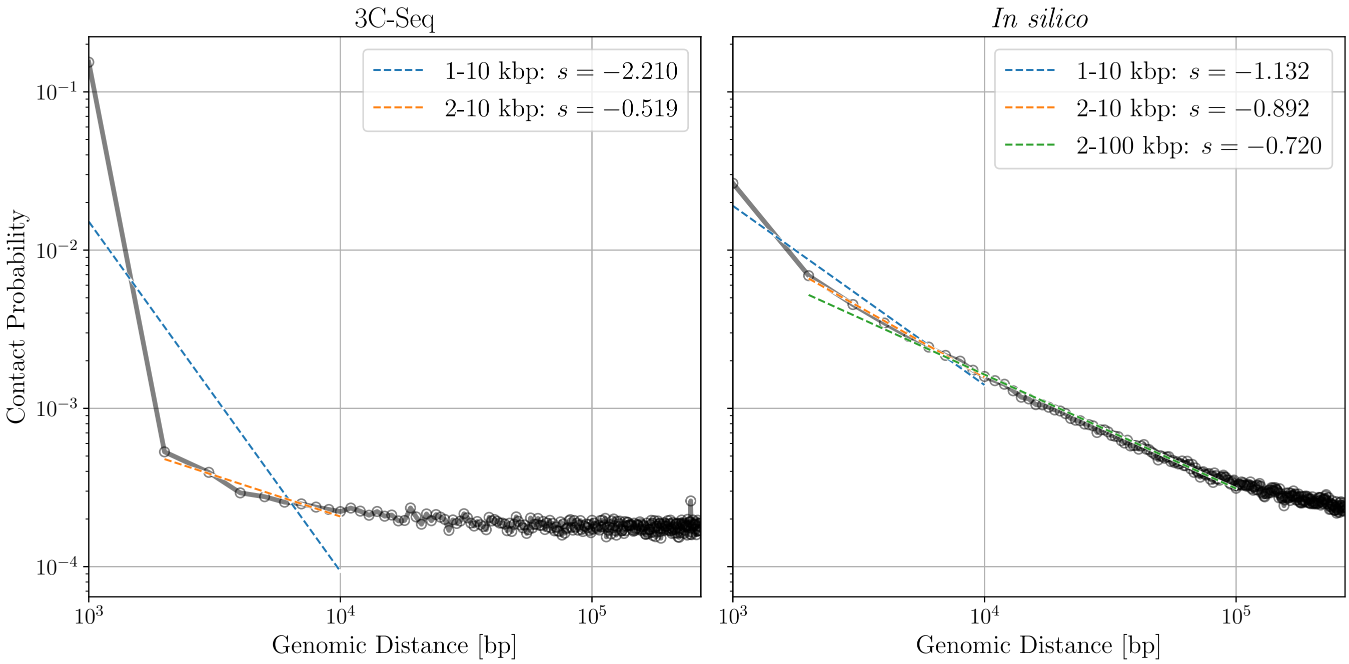

Figure S8. Contact laws for 3C-Seq and *in silico* maps: The interaction frequencies at a resolution of 1 kbp are plotted as a function of genomic distance. Fitting power laws of the form $P(x) \propto x^s$ to identical regions of the two datasets using a least-squares fit gave different values of s for each of the regions in both the 3C-Seq and *in silico* datasets, respectively. The power laws are super-imposed on the interaction frequencies and the corresponding values of s for each region are indicated in the legends.

REFERENCES

Durand, N. C., Robinson, J. T., Shamim, M. S., Machol, I., Mesirov, J. P., Lander, E. S., et al. (2016). Juicebox provides a visualization system for hi-c contact maps with unlimited zoom. *Cell Systems* 3, 99–101. doi:10.1016/j.cels.2015.07.012
